# Applying GenAI to Optimize Q-Matrix Construction for Cognitive Diagnostic Assessment in EFL Reading

**DOI:** 10.3390/jintelligence14050079

**Published:** 2026-05-05

**Authors:** Wenbo Du, Jiayi Shen, Xiaomei Ma

**Affiliations:** School of Foreign Studies, Xi’an Jiaotong University, Xi’an 710049, China; 546971977@stu.xjtu.edu.cn (J.S.); xiaomei@xjtu.edu.cn (X.M.)

**Keywords:** Q-matrix, generative artificial intelligence, cognitive diagnostic assessment, human–AI collaboration

## Abstract

Q-matrix construction is a foundational yet challenging step in cognitive diagnostic assessment (CDA), which is traditionally reliant on labor-intensive and subjective methods like expert judgment and verbal report analysis. This study explores the potential of generative artificial intelligence (GenAI) to optimize this critical process within the domain of EFL reading. By applying three GenAI models (DeepSeek-V3.2, Kimi 2.5, and Doubao 2.0), three purely GenAI-informed Q-matrices (Qmat-DS, Qmat-K, and Qmat-DB) were generated, and through expert revision, a human–AI collaborative Q-matrix (Qmat-DS-H) was obtained. These were compared with an expert-constructed Q-matrix (Qmat-E) and a student-derived Q-matrix (Qmat-S). Using a simulated dataset (*N* = 1000) and empirical response data from 1083 EFL learners on a diagnostic reading test, the psychometric performance of the six Q-matrices was estimated via the G-DINA model, ACDM model, and RRUM model. Results demonstrated that the human–AI collaborative Q-matrix consistently outperformed the other five Q-matrices, achieving the best absolute model-data fit, the highest classification accuracy, the most stable item parameters, and the most balanced attribute correlation structure. The purely GenAI-informed Q-matrices showed mixed results: there were some improvements in relative fit and slip stability compared to manually constructed Q-matrices, but variable absolute fit and attribute correlation patterns. The findings substantiate GenAI as a feasible pathway for enhancing the efficiency, consistency, and psychometric quality of Q-matrix construction. This study offers a preliminary framework for advancing CDA development, addressing a key methodological bottleneck in language assessment.

## 1. Introduction

Cognitive diagnostic assessments (CDAs) have advanced significantly in educational assessments over the past decades. Its strength lies in providing fine-grained diagnostic feedback to test-takers, which ultimately facilitates learning and instruction (Mei & Chen, 2022; C. Wang, 2024). In the field of language testing and assessment, CDA has been applied to various domains, including reading (e.g., H. Chen et al., 2023; Du & Ma, 2026), listening (e.g., Meng & Fu, 2023; Min & He, 2022), writing (e.g., He et al., 2021; Shi et al., 2024) and speaking (e.g., Liu et al., 2025). A typical CDA involves three steps: (1) defining cognitive attributes; (2) constructing a Q-matrix; and (3) psychometrical estimation via cognitive diagnostic models (CDMs). Cognitive attributes refer to a group of predefined subskills, knowledge, or strategies that a learner must possess or employ to successfully answer a test item (Leighton & Gierl, 2007; Nichols et al., 1995). Q-matrix is denoted as a binary incidence matrix that specifies the hypothesized relationship between test items and cognitive attributes, in which an entry of 1/0 indicates whether or not an item specifies a certain cognitive attribute (Rupp et al., 2010). With Q-matrix and the test-takers’ item responses as indicators, a family of psychometric models (CDMs) are designed to classify test-takers’ mastery status on specific cognitive attributes, thereby delivering detailed diagnostic feedback (Lee & Sawaki, 2009).

Among the three steps, the diagnostic precision of CDA fundamentally depends on the quality of its core component—the Q-matrix—which serves as the cognitive model that links observable item responses to latent attribute mastery. In current practice, however, the Q-matrix is predominantly constructed through expert judgment and/or learners’ verbal report, a process that is not only labor-intensive but also inherently subjective and prone to inconsistency (Du & Ma, 2021; Toprak & Cakir, 2021). The reliance on manual specification often results in two critical issues: (1) overspecification, where unnecessary attributes are included, and (2) under-specification, where essential attributes are omitted (Chiu, 2013; Rupp & Templin, 2008). These misspecifications may lead to model misfits (Mirzaei et al., 2020), low classification accuracy (Im & Corter, 2011) and unreliable diagnostic feedback (Toprak & Cakir, 2021).

Recent advances in generative artificial intelligence (GenAI), particularly large language models, offer a transformative opportunity to address these methodological challenges (Xi, 2025). GenAI demonstrates remarkable potential in redefining test constructs (Y. Wang & Meng, 2026; Xi, 2023), automated item generation (Aryadoust et al., 2024), automated scoring (Y. Kim, 2025), and providing personalized feedback (Mahapatra, 2024; Polakova & Ivenz, 2024). It also shows capabilities that closely parallel the cognitive analysis when developing the Q-matrix. First, GenAI is proficient in natural language processing and text parsing; thus, it can extract key information from item stems, identify question types, and detect subtle linguistic cues (Aryadoust et al., 2024). Second, it exhibits strong task decomposition and logical reasoning skills, enabling them to break down a test item into constituent elements (e.g., main claim, supporting details, implied conclusions) (Cai & Min, 2025; Xi, 2023). Third, GenAI can be prompted to follow explicit decision rules, and to produce structured binary outputs in a matrix format. This capacity for prompt-based coding process directly aligns with the binary coding task required for Q-matrix specification. Therefore, in the context of Q-matrix construction, a GenAI model can be instructed to simulate the cognitive analysis that humans would perform by identifying patterns in the item that correlate with the presence or absence of specific cognitive attributes. However, its potential for Q-matrix development in language assessment remains largely unexplored. To the best of our knowledge, no empirical study has investigated the feasibility of incorporating GenAI into Q-matrix construction. The present study aims to fill this gap by evaluating whether GenAI can produce Q-matrices that are not only efficient to generate but also statistically coherent and diagnostically accurate. The findings are expected to contribute a novel, scalable methodology to the CDA field and offer practical insights for developing more valid and reliable diagnostic assessments in language education.

## 2. Literature Review

### 2.1. Q-Matrix Specification

The Q-matrix was put forward by Tatsuoka (1985), which specifies the binary relationship between cognitive attributes and test items. As shown in Table 1, the columns consist of three attributes (A1–A3), which are dichotomously embedded in each item. For example, item 1 specifies A2 for an entry of 1, but does not measure A1 and A3. It also should be noted that item 2 and item 3 specify more than one attribute, indicating a complex loading structure referred to as within-item multidimensionality (Ravand & Baghaei, 2020).

Correct Q-matrix specification is crucial to the reliability and validity of CDA. Any misspecifications may largely decrease diagnostic accuracy (Kunina-Habenicht et al., 2012). Rupp and Templin (2008) demonstrated that misspecifications in the Q-matrix directly degrade parameter estimation and classification accuracy. Specifically, the incorrect omission of required attributes inflates slip parameters, while the spurious inclusion of non-required attributes leads to an underestimation of guessing parameters. Such inaccuracies can subsequently result in the misclassification of examinees, undermining the primary objective of CDA (Gao et al., 2017).

Beyond classification accuracy, Q-matrix misspecification may result in high attribute correlations (Aryadoust, 2021; Lee & Sawaki, 2009). If two or more attributes cannot be distinguishable, it is likely that they measure the same construct (Ravand & Robitzsch, 2018). In practice, these highly correlated attributes are combined into a composite attribute within the Q-matrix to mitigate the issue (Jang, 2009; A. Kim, 2015; Toprak & Cakir, 2021). However, such attempts may cause construct underrepresentation in real testing (Bachman & Palmer, 1996), which poses significant challenges for a fine-grained diagnosis (Rupp et al., 2010). Therefore, a valid Q-matrix is the core blueprint in CDA, whose specification issue cannot be overlooked.

### 2.2. Existing Approaches of Q-Matrix Construction

In existing CDA studies, Q-matrices are mainly constructed via expert judgment (e.g., H. Chen et al., 2023; A. Kim, 2015; Ravand & Robitzsch, 2018; Toprak & Cakir, 2021) and verbal report analysis (e.g., Du & Ma, 2021; Jang, 2009; Javidanmehr & Anani Sarab, 2019). While both methods have contributed valuable insights, each is associated with specific methodological limitations regarding subjectivity, inter-expert variability, and scalability.

Expert judgment requires a panel of at least five content experts to familiarize the definitions of cognitive attributes and then code the Q-matrix individually (Ravand & Baghaei, 2020). The coding agreement among experts is either calculated by Fleiss Kappa (Du & Ma, 2026; A. Kim, 2015) or following the coding results of the majority (H. Chen et al., 2023). When inconsistency occurs, several rounds of discussions are held among experts until consensus is reached (Jang, 2009). Expert judgment is valued for its direct leverage of domain expertise and theoretical grounding. The consensus-building process enhances the face and content validity of the Q-matrix (Ravand & Baghaei, 2020).

However, this approach is inherently resource-intensive, requiring significant time and coordination among highly specialized individuals (Qin & Guo, 2024). More critically, it is susceptible to subjectivity, where personal interpretations of attribute definitions or test items can lead to systematic misspecifications (i.e., overspecification or under-specification) (Cai & Min, 2025). The inter-expert variability, even among well-trained specialists, is well documented, with reported Fleiss Kappa values often falling in the moderate range (Du & Ma, 2021; Jang, 2009; A. Kim, 2015). This variability raises concerns about the replicability of the diagnostic results. Different expert panels may produce different Q-matrices for the same test, leading to inconsistent classifications. Furthermore, the lack of scalability is also a limitation. Assembling and coordinating a panel of experts for each new test is logistically prohibitive in large-scale or resource-constrained contexts.

In contrast, verbal report analysis (e.g., think-aloud protocols) offers a valuable bottom-up perspective by grounding the Q-matrix in the actual cognitive processes employed by learners (Jang, 2009). Verbal reports can reveal unexpected or overlooked cognitive attributes, thereby challenging and refining expert assumptions (Ravand & Baghaei, 2020). They provide empirical evidence of how test-takers actually interact with items, which can improve the authenticity of the Q-matrix.

Nevertheless, verbal reports have their own limitations. First, the validity of verbal reports can be compromised by learners’ metacognitive awareness, verbalization ability, and the potential for the think-aloud task itself to alter cognitive processing (Ericsson & Simon, 1993). Second, subjectivity remains an issue because coding the verbal protocols into the Q-matrix still requires human judgment, and different coders may interpret the same verbalizations differently (Leighton & Gierl, 2007). Third, inter-coder variability is also common, and is often comparable to or even higher than in expert judgment, because the verbal protocols are less structured (Jang, 2009). Finally, scalability is another critical drawback. Collecting, transcribing, and analyzing verbal reports from a sufficient number of learners (typically 5–20) is time-consuming and costly, making it impractical for large-scale test development (Bachman & Palmer, 1996). Consequently, while it provides crucial empirical insights, it is often used complementarily rather than as a standalone, definitive method for Q-matrix construction.

### 2.3. Q-Matrix Modification Extensions

To overcome misspecifications in Q-matrix, some empirical methods have been proposed, such as the generalization of the discrimination index (GDI) method (de la Torre & Chiu, 2016), the regularized latent class analysis (RLCA) (Y. Chen et al., 2017), iterative Q-Matrix validation methods (Terzi & de la Torre, 2018; Nájera et al., 2020) and, more recently, exploratory factor analysis (EFA) integrated with expert judgment (Cai & Min, 2025). These methods share a common conceptual logic. They treat an initially hypothesized Q-matrix as a provisional blueprint and iteratively revise it based on statistical patterns observed in examinees’ response data. As such, they are post hoc, data-driven, and statistical in nature. However, as Jang (2009) claimed, some modification methods can result in a less interpretable Q-matrix, which may violate the actual cognitive model’s specific underlying test domains (H. Chen et al., 2023). Even though these methods mark a crucial advancement towards a more evidence-based Q-matrix, their fundamental nature as post hoc optimization tools does not address the core inefficiency and subjectivity of the initial construction process. Similarly, Cai and Min (2025) found that EFA recovered fewer attributes than intended, conflating several theoretically distinct reading attributes into coarse-grained factors. This indicates that EFA can only reveal the psychometric dimensions that are actually measured, which may not align with the theoretical cognitive model.

Importantly, the GenAI-informed approach proposed in the present study differs fundamentally from the above empirical validation methods. Being generative in nature, GenAI develops a Q-matrix directly from the semantic content of test items and the definitions of cognitive attributes, without requiring an initial Q-matrix or response data. Therefore, while empirical validation methods address the correction of existing Q-matrices, GenAI addresses the construction of Q-matrices from scratch. The former is reactive and data-dependent, whereas the latter is proactive and content-dependent.

Based on the reviewed literature, constructing a valid and reliable Q-matrix remains a fundamental yet challenging bottleneck in CDA development. A critical gap, therefore, lies in the initial Q-matrix construction phase. There is a lack of a scalable, systematic, and theoretically informed method to generate a high-fidelity hypothesized Q-matrix prior to costly data collection and expert ratification. The recent advent of GenAI presents a paradigm-shifting opportunity to address this gap. GenAI can be strategically deployed to automate the cognitive task analysis that underpins Q-matrix specification.

To this end, this study aims to develop and evaluate a GenAI-informed framework for constructing and refining Q-matrix in the context of EFL reading diagnostic assessment. Specifically, it seeks to investigate whether and how GenAI can enhance the diagnostic accuracy, efficiency, and theoretical grounding of the Q-matrix, thereby mitigating the subjectivity and error inherent in traditional manual approaches. Two research questions are addressed:

1. To what extent can GenAI-informed Q-matrices achieve comparable or superior model-data fit to manually constructed Q-matrices?
2. To what extent can GenAI-informed Q-matrices outperform manually constructed Q-matrices in terms of classification accuracy and attribute correlation?

## 3. Method

### 3.1. Research Design and Hypothesis

This study adopted a comparative, cross-sectional design to evaluate the psychometric performance of six Q-matrices constructed through different methods: (1) experts’ Q-matrix (Qmat-E); (2) students’ Q-matrix (Qmat-S); (3) three GenAI-informed Q-matrices generated by DeepSeek (Qmat-DS), Kimi (Qmat-K), and Doubao (Qmat-DB); (4) one human–AI collaborative Q-matrix (Qmat-DS-H). All six Q-matrices were specified for the same diagnostic reading test and estimated using the same response dataset and the same cognitive diagnostic model. An additional simulated dataset was also generated using R program (version 4.5.1, R Core Team, Vienna, Austria) containing a similar sample size (*N* = 1000) and test length (20 items) to the empirical data for model robustness check. The guessing and slip parameters of the simulated 20 items were randomly generated within (0.05–0.15) as high quality (Ma & de la Torre, 2020). The diagnostic capacities were evaluated by model-data fit (both at the test level and item level) (RQ1), classification accuracy and attribute correlation (RQ2). Based on the reviewed literature, two hypotheses were formulated:

(1) The three purely GenAI-generated Q-matrices (Qmat-DS, Qmat-K, Qmat-DB) will demonstrate comparable and improved psychometric performance compared to two manually constructed Q-matrices (Qmat-E and Qmat S).
(2) The human–AI collaborative Q-matrix (Qmat-DS-H) will outperform all other Q-matrices across most psychometric indicators.

### 3.2. Instruments and Data Description

As stated earlier, two components are required for Q-matrix construction, i.e., cognitive attributes and test items. The cognitive attributes in this study were adopted from Du and Ma (2026), including six reading inferential attributes: A1, referential inference; A2, lexical inference; A3, temporal inference; A4, causative inference; A5, premise–conclusion inference; and A6, thematic inference; and two linguistic attributes: A7, understanding sentence’s literal meaning; and A8, understanding discourse’s literal meaning. The detailed definitions of the eight attributes can be seen in the work of Du and Ma (2026). All eight attributes were covered in an online diagnostic reading test for inferencing whose difficulty level was based on China’s Standards of English language ability (CSE): between CSE 5 and CSE 6. The diagnostic test comprised 20 items spanning four task formats: multiple-choice questions (*N* = 10), sentence ordering (*N* = 3), short-answer construction (*N* = 2), and title selection (*N* = 5). The test was administered online to a cohort of 1083 undergraduate students recruited from five universities in China. Each item was dichotomously scored (1 for a correct response; 0 otherwise), yielding a maximum total score of 20 points. Students’ test response data were then collected for Q-matrices validation purposes.

### 3.3. Q-Matrices Construction Procedure

The Qmat-E and Qmat-S were adapted from a previous study (see Du & Ma, 2026). Specifically, the Qmat-E was established through a structured expert judgment process. A panel of seven content experts in the EFL reading and CDA fields was convened. Each expert independently coded the Q-matrix after extensive training and discussion to ensure a shared understanding of the eight reading attributes. Their inter-rater agreement, calculated using Fleiss’ Kappa, was 0.569, indicating a moderate agreement (Landis & Koch, 1977). Following initial coding, a consensus-building discussion was held to resolve discrepancies. For example, the Fleiss’ Kappa of seven experts on item 6 was only 0.417. For this item, one expert coded A2 (lexical inference), four experts coded A4 (causative inference), one expert coded A6 (thematic inference), and four experts coded A7 (sentence literal meaning). During the discussion, each expert reviewed the item and stated their detailed coding process. Experts who coded A2 and A6 found that these were not core attributes this item targeted. Eventually, all experts agreed that item 6 specified A4 and A7. After all discrepancies were solved, we recalculated the Fleiss’ Kappa using the experts’ coding to quantify the final level of agreement. It increased from 0.569 to 0.833, reaching substantial agreement. Beyond inter-rater agreement, we also conducted a post hoc empirical validation using the general discrimination index (GDI) proposed by de la Torre and Chiu (2016). The GDI method was applied to the final Qmat-E using the simulated data. The GDI results indicated that the attribute specifications among 17 items in Qmat-E were empirically supported (GDI > 0.10), with only three item–attribute pairs (item 3, item 13, and item 20) showing marginal discrimination. This provides statistical evidence that the Q-matrix is largely consistent with the response patterns. After further content analysis of the three items by the experts, their attribute specifications aligned with the theoretical cognitive process. Therefore, the coding of these three items was retained. The final Qmat-E was obtained (see Table 2).

The Qmat-S was derived empirically from students’ cognitive processes to ground the Q-matrix in actual test-taking behaviors. A sample of 16 students representing three proficiency levels (low, intermediate, and high) based on their total reading scores completed a semi-structured self-report while taking the diagnostic test. They were required to write down their (1) initial thought, (2) answering process, and (3) use of strategies (Du & Ma, 2026). As noted by Ericsson and Simon (1993), verbal report samples typically range from 5 to 20 participants, as the goal is to achieve saturation of cognitive processes rather than statistical representativeness. Two trained researchers independently coded the data to identify the reading attributes that were actively employed for each item, resulting in Qmat-S (see Table 3). Their coding agreement, calculated by Cohen Kappa, was 0.89, reaching substantial agreement (Landis & Koch, 1977).

For the suitable GenAI engine, this study selected three widely used models in mainland China, i.e., the DeepSeek-V3.2 model, the Kimi 2.5 model, and the Doubao 2.0 model. Their proficiency in processing detailed, multi-part prompts containing item stems, options, and attribute definitions directly aligns with the requirement of simulating a structured, rule-based coding process. To illustrate, three Q-matrices were generated autonomously by the three models, tagged as Qmat-DS (DeepSeek-V3.2), Qmat-K (Kimi 2.5) and Qmat-DB (Doubao 2.0), to explore a fully algorithmic, theory-informed construction method. A detailed, structured prompt was engineered, providing three models with: (1) specific roles and target task backgrounds; (2) precise definitions and operational descriptions of the eight reading attributes; (3) the full text and options for each of the 20 test items; and (4) explicit instructions to output a binary Q-matrix with a justification for each coding decision. The full prompts, the diagnostic text paper, along with an output documentation example, can be found in the Supplementary Materials. The prompts for each item were as follows:

Prompt 1 (Role Specification): You are an expert specializing in cognitive diagnostic assessment (CDA) and language testing. Your task is to perform a rigorous cognitive analysis of an EFL diagnostic reading test and construct a corresponding Q-matrix. The test targets learners at approximately CSE 5 to CSE 6. Your analysis must be based solely on the provided materials and definitions, simulating the deductive reasoning process of a content expert.

Prompt 2 (Internalizing Attributes): Please review and internalize the following operational definitions of eight cognitive attributes. These definitions will serve as the exclusive coding framework for your analysis. Later, you are going to code which attribute or attribute combinations each test item measures.

Prompt 3 (Q-Matrix Coding Protocol): You will construct a binary Q-matrix. For each item, assign a value of ‘1’ if and only if mastery of the specific attribute or attribute combinations is theoretically necessary for a test-taker to answer the item correctly. Assign a value of ‘0’ if the attribute or attribute combinations are not necessary for a correct response.

Prompt 4 (Task Assigned): Now, please read Passage 1 carefully and answer item 1. You should briefly explain the cognitive processes required to answer this item correctly. Based on your answering process, code which attribute or attribute combinations item 1 measures. If you identify any new attributes, propose them with clear operational definitions and justify its necessity.

Based on the above coding prompts, the reasoning process and initial Q-matrices of the three GenAI models were captured. To ensure robustness, this procedure was repeated in three independent sessions with the same prompt across three models. Following the three generations, we applied a majority rule to determine the final GenAI-informed Q-matrix for each item–attribute pair:

(1) If all three generations coded an attribute as 0 or 1, that value was retained.
(2) If two of the three generations coded an attribute as 1 and one coded it as 0, the attribute was coded as 1 (indicating the attribute was required).
(3) If two of the three generations coded an attribute as 0 and one coded it as 1, the attribute was coded as 0 (indicating the attribute was not required).

To illustrate, among the three Q-matrices generated by the DeepSeek-V3.2 model, complete agreement occurred for 16 items (80%). For the remaining four items (item 6, item 8, item 13, and item 14), the above majority rule was applied. For example, if two of the three Q-matrices coded A4 and A8 for Item 6, while one coded A4 and A7, then A4 and A8 were retained for item 6. Discrepancies in other items were solved in the same way. The three final GenAI-generated Q-matrices (Qmat-DS, Qmat-K, and Qmat-DB) are shown in Table 4, Table 5 and Table 6, respectively.

The Qmat-DS-H was developed through an iterative hybrid protocol designed to synthesize AI efficiency with expert validation (see Table 7). The best fitting GenAI-generated Q-matrix (i.e., Qmat-DS) from the above session served as the initial draft. This draft was presented to two content experts who constructed Qmat-E, along with the GenAI-generated justifications for each coding decision. Experts were required to review each cell of the matrix, accepting entries they agreed with, and modifying or rejecting entries based on theoretical or contextual grounds not captured by the GenAI. This review was followed by a final consensus discussion focused specifically on the disputed entries. The resulting Qmat-DS-H represents a refined cognitive model that integrates data-driven AI hypothesizing with expert oversight and domain-specific nuance.

### 3.4. Q-Matrices Validation Procedure

To verify the validity and diagnostic utility of the six constructed Q-matrices, the G-DINA model (de la Torre, 2011), along with the ACDM model and RRUM model, was applied to estimate the simulated data and test-response data using the R packages G-DINA version 2.9.4 (Ma & de la Torre, 2020). The G-DINA model was selected for the following reasons. First, as a saturated model, the G-DINA subsumes many commonly used reduced CDMs as special cases, including the DINA, the DINO, the RRUM and the ACDM. Importantly, when evaluating Q-matrix validity, G-DINA imposes the fewest a priori assumptions about how attributes combine, allowing each item to be modeled with the most appropriate condensation rule. This flexibility makes G-DINA the preferred choice for Q-matrix validation studies, as it minimizes the risk of a model misspecification that could artifactually favor one Q-matrix over another (de la Torre, 2011; Ma & de la Torre, 2020). Second, because G-DINA can be constrained to yield simpler models, evaluating Q-matrices under G-DINA provides a stringent test: a Q-matrix that demonstrates superior fit under the most flexible model is unlikely to show contradictory results under more reduced models (Ma & de la Torre, 2020). To verify the robustness across different modeling assumptions, ACDM and RRUM were chosen: ACDM assumes that each attribute contributes additively to the probability of a correct response, with no interactions between attributes, while RRUM assumes a reduced, non-compensatory model where each attribute that is missing multiplicatively reduces the success probability (de la Torre, 2011).

To answer RQ1, six Q-matrices were separately fitted with the three CDMs. The model-data fit was checked both at the test level and item level. With regard to test-level fit, both absolute fit statistics (i.e., Max zr, Max zl) and relative fit statistics (i.e., Akaike’s Information Criterion, AIC; Bayesian Information Criterion, BIC; and −2 log-likelihood, −2LL) were examined. A non-significant adjusted *p*-value of Max zr and Max zl indicates good fit (J. Chen et al., 2013). For AIC, BIC, and −2LL, lower values denote a more optimal balance of model fit and parsimony. In terms of item-level fit, the heatmap plot showing the adjusted *p*-values for all item pairs were extracted. Misfitting item pairs occur if the adjusted *p*-value is less than .05 (Ma & de la Torre, 2020), and are tagged red in the plot. Meanwhile, the item parameter stability was also reported by computing standard errors for guessing and slip parameters. A more coherent Q-matrix yields parameter estimates with smaller standard errors on average, reflecting better model identification and less estimation uncertainty (de la Torre & Chiu, 2016). It should be noted that for the simulated dataset, only test-level data fit was checked, in that the simulated test items and sample had no practical meaning. Similarly, for ACDM and RRUM, only model-data fit results were reported, which are the most salient indices to determine whether the model fits the data well.

To answer RQ2, the classification accuracy of the four Q-matrices was inspected on both the test level and attribute level using P_(a)_ value. Test-level classification accuracy refers to the overall proportion of test-takers who are correctly classified. Attribute-level classification accuracy is denoted as to what extent test-takers can be correctly classified on each attribute (Johnson & Sinharay, 2018). Johnson and Sinharay (2018) proposed specific benchmarks for classification accuracy in diagnostic assessment: a test-level P_(a)_ value greater than .70 and an attribute-level P_(a)_ value greater than .80. These widely cited benchmarks were adopted in this study to evaluate the adequacy of the diagnostic classifications. To further testify whether the Q-matrices can yield a well-separated attribute structure, the tetrachoric correlations among attributes of six Q-matrices were computed. A more valid Q-matrix should produce attribute correlations that are moderate (reflecting related but distinct constructs) without extreme redundancy (r > 0.90) or excessive orthogonality (r < 0.20) (Rupp et al., 2010; von Davier & Lee, 2019).

## 4. Results

### 4.1. RQ1 Model–Data Fit Results Comparsion

#### 4.1.1. Test-Level Model Fit Statistics Across Six Q-Matrices

Test-level model fit statistics are essential to examine whether a Q-matrix is valid or not. The absolute and relative fit statistics of six Q-matrices on both a simulated dataset (*N* = 1000) and an empirical dataset (*N* = 1083) across three CDMs are shown in Table 8.

The absolute fit indices, assessed by the adjusted significance of the Max *z*(*r*) and Max *z*(*l*), revealed a clear hierarchy. As for simulated dataset, four Q-matrices (Qmat-S, Qmat-K, Qmat-DB and Qmat-DS-H) estimated by G-DINA showed good fit, indicated by non-significant adjusted *p*-values of Max *z*(*r*) and Max *z*(*l*). However, the adjusted *p*-values of Qmat-S, Qmat-K and Qmat-DB were 1.00, indicating oversimplified models and potential underrepresentation (de la Torre & Chiu, 2016). Both Qmat-E and Qmat-DS demonstrated misfit, with adjusted *p*-values below .05. As for ACDM and RRUM, both Qmat-S and Qmat-DS-H demonstrated better fit, while other Q-matrices showed significant misfit (adj-*p* < .01).

With regard to empirical dataset, when estimated by G-DINA, Qmat-E and Qmat-S showed significant misfit, with adjusted *p*-values below .00 for both statistics. Qmat-K and Qmat-DB similarly exhibited significant misfit (adj-*p* < .01). In contrast, Qmat-DS showed improved but marginal fit, with adjusted *p*-values being .13 and .20 respectively. Notably, the model anchored by the human–AI collaborative Qmat-DS-H demonstrated the best absolute fit, with non-significant adjusted *p*-values for both Max *z*(*r*) (.47) and Max *z*(*l*) (.66), indicating decent model–data fit. For ACDM and RRUM, even though no Q-matrices reached acceptable absolute fit, Qmat-DS-H demonstrated improved performance under ACDM with the lowest value of Max zr (4.50) and Max zl (4.46). Qmat-E showed the lowest value of Max zr (5.04) under RRUM, while Qmat-DB depicted the lowest value on Max zl (5.34).

The relative fit indices presented a more nuanced picture. For the simulated dataset, when estimated by G-DINA, Qmat-K depicted the lowest AIC value (23,503.60), followed by Qmat-DB (23,511.64) and Qmat-DS-H (23,520.47). The lowest BIC value was observed for Qmat-DB (25,312.42), followed by Qmat-S (25,313.32) and Qmat-E (25,255.74). Qmat-DS yielded the best performance on −2LL (22,606.22), followed by Qmat-DS-H (22,706.48) and Qmat-K (22,745.60). Qmat-E and Qmat-S showed the two highest values on −2LL. When estimated by ACDM, Qmat-S showed the lowest value across the three indices, while Qmat-DS-H yielded a comparable performance with Qmat-S. The three GenAI-informed Q-matrices showed comparable fit with Qmat-E. Similarly, under RRUM, Qmat-DS-H demonstrated the lowest value on AIC (23,586.07) and −2LL (22,926.06), and the second lowest value on BIC compared to Qmat-S.

Regarding the empirical dataset, under G-DINA, the lowest AIC value was observed for Qmat-DB (25,305.49), followed by Qmat-S (25,333.07) and Qmat-K (25,360.40), while Qmat-E yielded the lowest value on BIC (27,070.17), followed by Qmat-DB (27,135.56) and Qmat-S (27,143.19). Qmat-DS-H yielded a very similar AIC (25,406.04) and a lower BIC (27,435.57) than Qmat-DS. For −2LL statistics, the Qmat-DS demonstrated the lowest value, followed by Qmat-DB, while Qmat-DS-H showed a comparable performance with Qmat-DB and Qmat-K. The Qmat-E and Qmat-S showed the least favorable values on −2LL. With regard to ACDM, Qmat-DS-H yielded the lowest value on AIC (25,466.52), while three GenAI-informed Q-matrices showed improved and comparable results with Qmat-E and Qmat-S. Similar results were also found in RRUM. Although Qmat-E yielded the lowest value on BIC (27,059.89), Qmat-DS-H showed better performance on −2LL, followed by the three GenAI-informed Q-matrices. Taken together, the three GenAI-informed Q-matrices (Qmat-DS, Qmat-K, and Qmat-DB) performed better on relative fit statistics across three selected CDMs in both datasets, while human–AI collaborative Q-matrix (Qmat-DS-H) largely improved the absolute fit compared to manually constructed Q-matrices (Qmat-E and Qmat-S) and GenAI-informed Q-matrices. Additionally, it showed robust performance across different CDMs on both datasets.

#### 4.1.2. Item-Level Fit Results Across Six Q-Matrices

**Figure 1:**
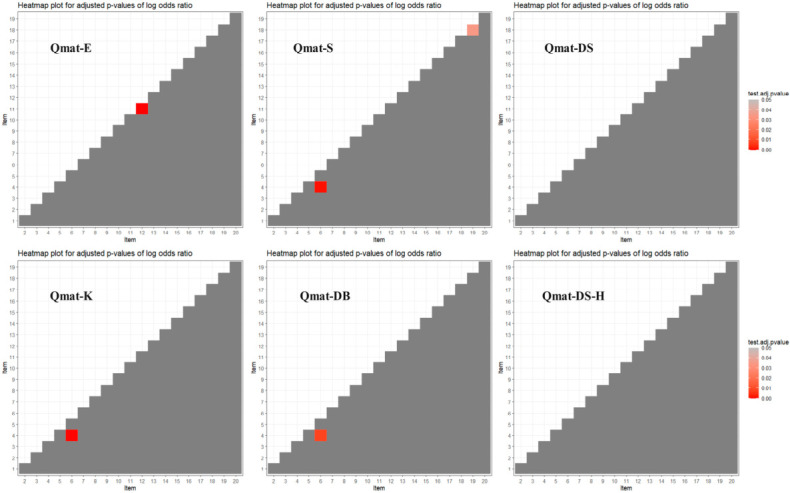
Heatmap plot for all item pairs of six Q-matrices.

The heatmap plot for all item pairs of the six Q-matrices is shown in Figure 1.

In the plot, misfitting item pairs could be found in two manually constructed Q-matrices and two GenAI-informed Q-matrices. Specifically, one item pair (item 11 and item 12) was misfit in Qmat-E and two item pairs (item 4 and item 6; item 18 and item 19) were misfit in Qmat-S. In Qmat-K and Qmat-DB, the same item pair (item 4 and item 6) was misfit. In contrast, all item pairs in Qmat-DS and Qmat-DS-H were well fitted to the data, showing satisfactory item-level fit.

#### 4.1.3. Item Parameters Stability Across Six Q-Matrices

The guessing (*g*) and slip(*s*) parameters of 20 items, along with their standard errors (SE), as estimated by the six Q-matrices, are presented in Table 9.

Among the six Q-matrices, Qmat-DS-H demonstrated the lowest mean standard error on both guessing (mean SE = 0.027) and slip (mean SE = 0.027) parameters, indicating a more stable parameter estimate. The three GenAI-informed Q-matrices (Qmat-DS, Qmat-K, and Qmat-DB) produced better estimates on the slip parameter, with mean standard errors ranging from 0.021 to 0.028. However, their estimates on the guessing parameter were less stable, reflected in the higher mean standard errors ranging from 0.039 to 0.047. The two manually constructed Q-matrices (Qmat-E and Qmat-S) demonstrated better estimates on the guessing parameter, with the mean standard errors being 0.040 and 0.036 respectively. Their estimates on the slip parameter, however, were worse than the GenAI-informed Q-matrices. Taken together, the Qmat-DS-H provided better model identification and less estimation uncertainty than its rival Q-matrices.

### 4.2. RQ2 Classification Accuracy and Attribute Correaltion Comparison

#### 4.2.1. Classification Accuracy Across Six Q-Matrices

The classification accuracy indices of the six Q-matrices at the test level and attribute level are reported in Table 10.

A critical benchmark for acceptable test-level classification accuracy, as per Johnson and Sinharay (2018), is P_(a)_ > .70. Among the six Q-matrices, only Qmat-DS-H (0.712) and Qmat-DB (0.709) met this criterion, followed by Qmat-S (0.696). The Qmat-DS was marginally below, at 0.692, followed by Qmat-K (0.674). Notably, the Qmat-E (0.570) yielded the lowest test-level classification accuracy, substantially below the acceptable benchmark.

At the attribute level, where P_(a)_ > .80 is considered acceptable, all six Q-matrices performed adequately for most attributes. However, clear differences emerged. Qmat-DS-H demonstrated consistently high attribute-level accuracy across all eight attributes, with six attributes (A2, A3, A4, A5, A6, A7) exceeding .90 and the highest accuracy reaching 0.981 (A6). Its performance on A1 (0.880) and A8 (0.898) was slightly lower but still well above the 0.80 threshold. Qmat-DB and Qmat-S showed strong performance on most attributes, with five attributes above 0.90. Qmat-DS and Qmat-K yielded comparable results, each with four attributes above 0.90. While Qmat-E also yielded acceptable attribute-level accuracy for most attributes, it demonstrated the lowest value among all Q-matrices. In summary, Qmat-DS-H outperformed its rival Q-matrices at both the test level and attribute level on classification accuracy.

#### 4.2.2. Summary of Attribute Correlation Across Six Q-Matrices

For ease of interpretation, the number of attribute pairs falling into three correlation ranges (i.e., high > 0.70; intermediate 0.20–0.70; and low < 0.20) was counted and is summarized in Table 11. Full attribute correlation matrices for all six Q-matrices are provided in Appendix A.

Following common practice in CDA (Rupp et al., 2010; von Davier & Lee, 2019), correlations in the range of 0.20–0.70 indicate meaningful but distinct relationships among attributes, whereas correlations above 0.70 suggest potential redundancy, and correlations below 0.20 indicate weak or negligible associations.

Notably, no Q-matrix produced any high correlation above 0.70, indicating that none of the attribute pairs suffered from severe redundancy. However, substantial differences emerged in the distribution of intermediate and low correlations. Qmat-DS-H exhibited the most intermediate correlations (24 out of 28 pairs, 85.7%) and the fewest low correlations (4 pairs, 14.3%). This suggests that attributes in Qmat-DS-H are consistently and moderately interrelated, which is a strong enough interrelation to reflect a coherent cognitive construct but not so strong as to be indistinguishable (Toprak & Cakir, 2021).

In contrast, the three GenAI-informed Q-matrices showed more variable patterns. Qmat-K demonstrated a high proportion of intermediate correlations (19 pairs, 67.9%) and relatively few low correlations (9 pairs, 32.1%), indicating a reasonably coherent structure. Qmat-DS (10 intermediate, 18 low) and Qmat-DB (6 intermediate, 22 low) displayed weaker inter-attribute associations, with the majority of correlations falling into the low range. With regard to two manually constructed Q-matrices, Qmat-S similarly showed a predominance of low correlations (20 pairs, 71.4%), while Qmat-E fell in the middle, with equal numbers of intermediate and low correlations.

In summary, the attribute correlation results indicate that Qmat-DS-H yields the most structurally coherent attribute structure, with a more balanced inter-attribute relationship. This finding complements the model-fit and classification accuracy results, further supporting the advantage of the human–AI collaborative approach.

## 5. Discussion

### 5.1. Advantages of GenAI-Informed Q-Matrix Construction

The findings of this study offer preliminary evidence suggesting that integrating GenAI into the Q-matrix construction may offer certain benefits. The expert constructed Q-matrix (Qmat-E), while theoretically grounded, demonstrated relatively weaker psychometric performance, with significant model–data misfit (adj *p*-values of Max *zr* and *zl* < .05) on both simulated and empirical dataset across three CDMs and the lowest test-level classification accuracy (.570), failing to meet the 0.70 benchmark (Johnson & Sinharay, 2018). This aligns with longstanding critiques of the subjectivity and inconsistency of sole reliance on expert panels (Cai & Min, 2025; H. Chen et al., 2023; Ravand & Baghaei, 2020). The student-derived Q-matrix (Qmat-S), although showing unacceptable absolute fit on the empirical dataset, performed better on the simulated dataset across three CDMs, especially in two relative fit indices (AIC and BIC), indicating the importance of student’s cognitive process in Q-matrix construction (Alderson et al., 2015; Leighton & Gierl, 2007).

The three GenAI-informed Q-matrices (Qmat-DS, Qmat-K, and Qmat-DB) demonstrated some improvements in specific fit indices. For instance, on both simulated and empirical dataset across three CDMs, all three Q-matrices showed lower −2LL values and, in some cases, lower AIC or BIC values, compared to two manually constructed Q-matrices, suggesting a potentially better balance between fit and parsimony (de la Torre & Chiu, 2016). However, their absolute fit remained problematic. On the simulated dataset, Qmat-DS showed significant misfit across three CDMs, while Qmat-K and Qmat-S demonstrated an overly ideal performance, with adj-*p* being 1.00 when estimated by G-DINA, an indication of underrepresentation issues (de la Torre & Chiu, 2016). On the empirical dataset, only Qmat-DS reached marginal non-significance (adj-*p* = .13 and .20) under G-DINA, while Qmat-K and Qmat-DB showed significant misfit across the three models. This indicates that fully automated GenAI generation, at least with the current prompts and models, may not consistently yield acceptable model–data fit without human oversight. In contrast, Qmat-DS-H achieved a more robust absolute fit on both simulated and empirical datasets across different CDMs. While its AIC is slightly higher than that of Qmat-S and Qmat-DB in most cases, this trade-off is acceptable because absolute fit is the primary criterion for Q-matrix validity (Ma & de la Torre, 2020). A direct comparison of the six Q-matrices (Table 2, Table 3, Table 4, Table 5, Table 6 and Table 7) revealed that Qmat-DS-H reduced overly complex, multi-attribute codings, as shown in Qmat-DS, which may lead to poor attribute discrimination (Madison & Bradshaw, 2015). This translated into superior absolute model–data fit, a stark contrast to the significant misfit of the purely human-derived Q-matrices (Qmat-E and Qmat-S).

Moreover, the three GenAI-informed Q-matrices demonstrated variable performance on item parameter stability. They produced more stable slip parameter estimates (mean SE from 0.021 to 0.028) compared to the manually constructed Q-matrices, but their guessing parameter estimates were less stable (mean SE from 0.039 to 0.047). This suggests that while GenAI may capture certain response patterns well, it may also introduce uncertainty in model estimation (Aryadoust et al., 2024).

Furthermore, the GenAI approach offers potential advantages in terms of scalability, consistency, and efficiency (Chuang & Yan, 2025). Unlike assembling and training a panel of experts and students, which require weeks of coordination and discussion, the GenAI model can generate a complete, logically justified Q-matrix prototype in a matter of minutes. This efficiency does not come at the cost of replicability as the same prompt and model will produce an identical analysis, eliminating the inter-panel variability that plagues expert judgment (Cai & Min, 2025). GenAI-informed Q-matrix construction directly responds to the call for more efficient and objective methods in test development, a domain in which GenAI has already shown promise in automated item generation (Aryadoust et al., 2024; Lin & Chen, 2024). The present study extends this application upstream to the critical stage of CDA modeling, an area that is less explored in current GenAI studies, which predominantly focus on automated scoring, AI feedback, and item generation (Chuang & Yan, 2025; Y. Kim, 2025; Mahapatra, 2024).

However, GenAI-informed Q-matrices also revealed the limitations of a purely AI-driven approach. While their test-level and item-level psychometric performances were comparable or superior to Qmat-E and Qmat-S, potential overspecification issues occur. In total, we found that five out of 20 items (25%) contained at least one over-specified attribute in Qmat-DS, while no substantial overspecification was found in Qmat-K and Qmat-DB. For Qmat-DS, item 17 was coded with six attributes, which may violate the actual cognitive process required for that item. This suggests that the GenAI-informed Q-matrix may occasionally reflect patterns that are not fully aligned with the linguistic complexity, the curricular context, or the precise pedagogical intent behind a test item (Aryadoust et al., 2024; Xi, 2023). Moreover, if we compare the psychometric diagnostics between Qmat-DS and Qmat-DS-H, we found that the test-level model-fit improved after removing over-specified attributes. For item-level statistics, among the five over-specified items, the guessing and slip parameters of four items (item 12, item 13, item 15, and item 17) decreased. At the attribute level, the classification accuracy of A5, which was most frequently over-specified, increased from 0.870 to 0.917. These findings indicates that over-specified attributes added unnecessary model complexity without explanatory benefit (Ma & de la Torre, 2020). A GenAI model, trained on vast general corpora, may not fully grasp the specific cognitive demands intended for learners at the CSE 5–6 level, potentially leading to oversimplifications or misinterpretations that a domain expert would catch. Therefore, GenAI overspecification could degrade model parsimony and can disproportionately harm classification accuracy for the affected attributes.

### 5.2. Necessity of Human–AI Collaboration in Q-Matrix Construction

A more consistent and robust performance across most psychometric indicators was observed for the human–AI collaborative Q-matrix (Qmat-DS-H). Qmat-DS-H achieved the best absolute model–data fit on both datasets across three CDMs, the highest test-level classification accuracy (0.712), and strong attribute-level classification accuracy (six attributes above 0.90). In addition, it exhibited the lowest mean standard errors for both guessing (0.027) and slip (0.017) parameters, indicating better parameter stability and lower estimation uncertainty. In terms of attribute correlations, Qmat-DS-H also showed the most balanced structure, with 24 out of 28 attribute pairs falling into the intermediate range (0.20–0.70), suggesting a coherent yet differentiated attribute structure.

These findings suggest that neither the purely manual nor the fully automated GenAI approaches alone were sufficient. Instead, a synergistic process, in which the GenAI generated an initial Q-matrix that experts subsequently reviewed and refined, appears to yield better results. Human–AI collaboration appears to mitigate the core weaknesses of each method in isolation (Jiang et al., 2024). The GenAI-generated matrix provides a structured, data-driven starting point, which experts can then evaluate against their domain knowledge. This concurs with a previous study on item distractor development (Y. Wang & Meng, 2026). In our case, where Qmat-DS showed potential overspecification (e.g., coding six attributes to an item), the experts were able to remove irrelevant attributes (A4, causative inference; A5, premise–conclusion inference; and A6, thematic inference) based on content analysis and domain knowledge. This transforms the expert’s role from primary coder to validator and calibrator, which may be a more efficient use of scarce human expertise (Aryadoust et al., 2024; Y. Kim, 2025; Xi, 2025).

The item-level fit results further support the advantage of the human–AI collaborative approach. While Qmat-DS and Qmat-DS-H both showed no misfit item pairs in the heatmap plot, the purely GenAI-informed Qmat-K and Qmat-DB each contained at least one misfit item pair (items 4 and 6). The manually constructed Q-matrices also showed misfitting pairs, which is consistent with findings from previous studies (He et al., 2022; Shi et al., 2024). Therefore, the combination of GenAI generation and expert revision may lead to more consistent item-level performance.

Additionally, the attribute correlation patterns provide complementary evidence. Different from previous studies, which demonstrated high attribute correlations (Lee & Sawaki, 2009; Ravand, 2016; Toprak & Cakir, 2021), Qmat-DS-H exhibited the most intermediate correlations, indicating that the attributes are neither redundant nor unrelated. In contrast, Qmat-DS and Qmat-DB showed predominantly low correlations (18 and 22 low pairs, respectively), which may indicate under-specification or weak attribute differentiation (Cai & Min, 2025; Jang, 2009). Qmat-S also showed many low correlations (20 pairs), while Qmat-E fell in the middle. These results suggest that the human–AI collaborative process yields a more balanced and theoretically meaningful attribute structure.

In summary, the findings of this study provide preliminary support for a “human-centered quality control system” advocated by Chuang and Yan (2025). They caution that for local testing contexts, resource constraints are a major barrier to GenAI adoption. The human–AI collaborative approach, which applies open-source GenAI models (such as DeepSeek, Kimi, and Doubao) to generate an initial Q-matrix, followed by expert review and revision, appears to be a feasible and effective method, at least for this specific diagnostic reading test and learner population. To clarify, GenAI is not merely a tool for automating existing tasks (Y. Kim, 2025) but a partner that can redefine the Q-matrix construction workflow. However, its full potential is only unlocked through deliberate collaboration with human expertise, which ensures that the final cognitive model is not only statistically sound but also theoretically meaningful and pedagogically valid (Cai & Min, 2025). It is also important to note that these findings are exploratory and based on a single dataset. Future research is needed to replicate the workflow with different tests, populations, and GenAI models before any strong claims can be made about generalizability.

### 5.3. Implications for Development of Diagnostic Assessments

The findings of this study also carry several methodological and practical implications for researchers and practitioners involved in developing diagnostic assessments. Methodologically, this study introduces a replicable workflow for GenAI-informed Q-matrix construction that can be adopted by other researchers. The workflow comprises: (1) defining cognitive attributes and drafting detailed prompts; (2) generating an initial Q-matrix using one or more GenAI models with repeated runs and majority rule; (3) having domain experts review and revise the GenAI-generated Q-matrix; and (4) validating the final Q-matrix using several psychometric criteria. This workflow addresses the reproducibility crisis often associated with qualitative judgment-based methods (Cai & Min, 2025; Javidanmehr & Anani Sarab, 2019). Practically, for language test developers and CDA practitioners, the human–AI collaborative approach offers a scalable and cost-effective alternative to traditional expert-panel or think-aloud methods (Aryadoust et al., 2024; Y. Kim, 2025). In contexts where assembling a panel of 5–7 content experts is logistically or financially prohibitive, a single expert (or a small team) can leverage GenAI to produce a defensible Q-matrix with acceptable psychometric properties. What might take weeks of expert discussion can be reduced to a few hours of prompt design, GenAI generation, and targeted expert review (Y. Wang & Meng, 2026; Xi, 2023). This efficiency could lower the barrier to implementing CDA in under-resourced educational settings, including K-12 schools, community colleges, and large-scale online learning environments.

## 6. Conclusions

This study investigated the potential of GenAI, specifically the DeepSeek-V3.2 model, the Kimi 2.5 model and Doubao 2.0 model, to optimize the construction of Q-matrices for cognitive diagnostic assessment in EFL reading. The findings offer preliminary evidence that GenAI may contribute to Q-matrix construction to some degree, while also highlighting the indispensable role of human expertise in refining and ensuring the linguistic interpretability of the Q-matrix.

Among the six Q-matrices compared, the human–AI collaborative Q-matrix (Qmat-DS-H) showed the most consistent psychometric performance, which corroborated hypothesis two. It achieved the best absolute model–data fit on both simulated and empirical dataset across three CDMs, showed the highest test-level classification accuracy, and maintained high attribute-level classification accuracy across all eight attributes. In addition, Qmat-DS-H exhibited better item parameter stability, and the most balanced attribute correlation structure. The three purely GenAI-informed Q-matrices (Qmat-DS, Qmat-K, and Qmat-DB) demonstrated certain improvements over the manually constructed Q-matrices in relative fit indices and in slip parameter stability. However, they varied considerably in attribute correlation patterns, potentially indicating attribute misspecification. As for the two manually constructed Q-matrices, the performance of Qmat-S surpassed that of Qmat-E, yet was slightly inferior to the GenAI-informed Q-matrices. These observations lend credence to hypothesis one. The above findings suggest that to some extent, the human–AI collaborative approach may not only mitigate the subjectivity inherent in the manual approach but could also address the uncertainty and inconsistency shown in purely GenAI automated methods.

Despite these promising results, several limitations must be acknowledged. First, due to accessibility issues, the three selected GenAI models were all Chinese-developed models. The task performance, biases, and reasoning patterns may vary across different large language models (e.g., GPT-4, Claude, Gemini). Future studies should employ other GenAI engines to compare their efficacy, consistency, and potential idiosyncrasies in Q-matrix construction. Second, all analyses were estimated by three selected CDMs (i.e., G-DINA, ACDM, and RRUM) under G-DINA framework on a simulated dataset and an empirical dataset from a diagnostic reading test focusing on reading and inferential skills. Consequently, the observed advantages of the GenAI-informed Q-matrices have not yet been demonstrated across other CDMs, reading tests, learner populations, or proficiency levels. The present findings, therefore, should be interpreted as preliminary evidence supporting the feasibility of GenAI-informed Q-matrix construction, rather than as a universally validated framework. Future research should replicate the proposed workflow using alternative CDMs and diagnostic reading assessments, with more diverse EFL samples and proficiency levels. Finally, this study was confined to a predefined set of eight reading attributes. The ability of GenAI to identify, define, or suggest novel cognitive attributes that may not be covered by existing theoretical frameworks warrants exploration. Future research could task GenAI with a more open-ended cognitive analysis to examine its potential for theory refinement.

## Figures and Tables

**Table 1:** An example of a Q-matrix.

Item	A1	A2	A3
Item1	0	1	0
Item2	1	1	0
Item3	1	1	1

**Table 2:** Experts’ Q-matrix.

Item	A1	A2	A3	A4	A5	A6	A7	A8	Item	A1	A2	A3	A4	A5	A6	A7	A8
Item 1	0	1	0	0	0	0	1	0	Item 11	0	0	0	0	0	1	0	1
Item 2	1	0	0	0	0	0	1	0	Item 12	0	0	0	0	0	1	0	1
Item 3	1	0	0	0	0	0	0	1	Item 13	0	0	0	0	0	1	0	1
Item 4	1	0	0	0	0	0	1	0	Item 14	0	0	0	0	0	1	0	1
Item 5	0	0	0	1	0	0	1	0	Item 15	0	0	0	0	0	1	0	1
Item 6	0	0	0	1	0	0	1	0	Item 16	0	0	1	0	0	0	0	1
Item 7	0	0	0	0	1	0	0	1	Item 17	0	0	1	1	0	0	0	1
Item 8	0	0	0	1	0	0	0	1	Item 18	0	0	1	0	0	0	0	1
Item 9	0	0	0	0	1	0	0	1	Item 19	0	1	0	0	0	0	0	1
Item 10	0	0	0	0	1	0	0	1	Item 20	0	1	0	0	0	0	0	1

Note. A1: referential inference; A2: lexical inference; A3: temporal inference; A4: causative inference; A5: premise–conclusion inference; A6: thematic inference; A7: understanding sentence’s literal meaning; A8: understanding discourse’s literal meaning.

**Table 3:** Students’ Q-matrix.

Item	A1	A2	A3	A4	A5	A6	A7	A8	Item	A1	A2	A3	A4	A5	A6	A7	A8
Item 1	0	1	0	0	0	0	1	0	Item 11	0	0	0	0	0	1	1	1
Item 2	1	0	0	0	0	0	1	0	Item 12	0	0	0	0	0	1	1	1
Item 3	1	0	0	0	0	0	1	1	Item 13	0	0	0	0	0	1	1	1
Item 4	1	0	0	0	0	0	1	1	Item 14	0	0	0	0	0	1	1	1
Item 5	0	0	0	1	0	0	1	0	Item 15	0	0	0	0	0	1	1	1
Item 6	0	0	0	1	0	0	1	0	Item 16	0	0	1	0	0	0	1	0
Item 7	0	0	0	0	1	0	0	1	Item 17	0	0	1	0	0	0	1	0
Item 8	0	0	0	1	0	0	1	0	Item 18	0	0	1	0	0	0	1	0
Item 9	0	0	0	0	1	0	0	1	Item 19	0	1	0	0	0	0	1	0
Item 10	0	0	0	0	1	0	0	1	Item 20	0	1	0	0	0	0	1	0

**Table 4:** DeepSeek-informed Q-matrix.

Item	A1	A2	A3	A4	A5	A6	A7	A8	Item	A1	A2	A3	A4	A5	A6	A7	A8
Item 1	0	1	0	0	0	0	1	0	Item 11	0	0	0	0	1	1	1	1
Item 2	1	0	0	0	0	0	0	1	Item 12	0	0	0	0	1	1	1	1
Item 3	1	0	0	0	0	0	0	1	Item 13	0	0	0	0	1	1	1	1
Item 4	1	0	0	0	0	0	0	1	Item 14	0	0	0	0	0	1	1	1
Item 5	0	0	0	0	1	0	1	0	Item 15	0	0	0	0	1	1	1	1
Item 6	0	0	0	1	0	0	0	1	Item 16	1	0	0	0	0	1	1	1
Item 7	1	0	0	0	1	0	0	0	Item 17	0	0	1	1	1	1	1	1
Item 8	0	0	0	0	0	1	0	1	Item 18	0	0	1	1	0	0	1	1
Item 9	0	0	0	1	0	0	0	1	Item 19	0	1	0	0	1	0	1	1
Item 10	0	0	0	1	1	0	0	1	Item 20	0	1	0	0	1	0	1	1

**Table 5:** Kimi-informed Q-matrix.

Item	A1	A2	A3	A4	A5	A6	A7	A8	Item	A1	A2	A3	A4	A5	A6	A7	A8
Item 1	0	1	0	0	0	0	1	0	Item 11	0	0	0	0	0	1	1	1
Item 2	1	0	0	0	0	0	1	0	Item 12	0	0	0	0	0	1	1	1
Item 3	1	0	0	0	0	0	1	0	Item 13	0	0	0	0	0	1	1	1
Item 4	1	0	0	0	0	0	1	0	Item 14	0	0	0	0	0	1	1	1
Item 5	0	0	0	1	0	0	1	0	Item 15	0	0	0	0	0	1	1	1
Item 6	0	0	0	1	0	0	0	1	Item 16	0	0	1	0	0	0	0	1
Item 7	0	0	1	0	0	0	0	1	Item 17	0	0	1	1	0	0	0	1
Item 8	0	0	0	1	0	0	0	1	Item 18	0	0	1	1	0	0	0	1
Item 9	0	0	0	0	1	0	0	1	Item 19	0	1	0	0	0	0	1	1
Item 10	0	0	0	0	1	0	0	1	Item 20	0	1	0	1	0	0	1	1

**Table 6:** Doubao-informed Q-matrix.

Item	A1	A2	A3	A4	A5	A6	A7	A8	Item	A1	A2	A3	A4	A5	A6	A7	A8
Item 1	0	1	0	0	0	0	1	0	Item 11	0	0	0	0	0	1	1	1
Item 2	1	0	0	0	0	0	1	0	Item 12	0	0	0	0	0	1	1	1
Item 3	1	0	0	0	0	0	1	0	Item 13	0	0	0	0	0	1	1	1
Item 4	1	0	0	0	0	0	1	0	Item 14	0	0	0	0	0	1	1	1
Item 5	0	0	0	1	0	0	1	0	Item 15	0	0	0	0	0	1	1	1
Item 6	0	0	0	1	0	0	1	0	Item 16	0	0	1	0	0	0	1	1
Item 7	0	0	0	0	1	0	1	0	Item 17	0	0	0	1	0	0	1	1
Item 8	0	0	0	1	0	0	1	0	Item 18	0	0	1	0	0	0	1	1
Item 9	0	0	0	0	1	0	1	0	Item 19	0	1	0	0	0	0	1	0
Item 10	0	0	0	0	1	0	1	0	Item 20	0	1	0	0	0	0	1	0

**Table 7:** Human–AI collaborative Q-matrix.

Item	A1	A2	A3	A4	A5	A6	A7	A8	Item	A1	A2	A3	A4	A5	A6	A7	A8
Item 1	0	1	0	0	0	0	1	0	Item 11	0	0	0	0	0	1	1	1
Item 2	1	0	0	0	0	0	0	1	Item 12	0	0	0	0	0	1	1	1
Item 3	1	0	0	0	0	0	0	1	Item 13	0	0	0	0	0	1	1	1
Item 4	1	0	0	0	0	0	0	1	Item 14	0	0	0	0	0	1	1	1
Item 5	0	0	0	0	1	0	1	0	Item 15	0	0	0	0	0	1	1	1
Item 6	0	0	0	1	0	0	0	1	Item 16	0	0	1	0	0	0	1	1
Item 7	1	0	0	0	1	0	0	0	Item 17	0	0	1	0	0	0	1	1
Item 8	0	0	0	1	0	0	0	1	Item 18	0	0	1	1	0	0	1	1
Item 9	0	0	0	1	0	0	0	1	Item 19	0	1	0	0	1	0	1	1
Item 10	0	0	0	1	1	0	0	1	Item 20	0	1	0	0	1	0	1	1

Note. The shadowed entries are the human-revised Q-matrix.

**Table 8:** Test-level model fit statistics of six Q-matrices across three CDMs.

Q-Matrices	Dataset	Model	Max zr (adj-*p*)	Max zl (adj-*p*)	AIC	BIC	−2LL
Qmat-E	Empirical dataset (*N* = 1083)	G-DINA	4.87(0.00)	5.38(0.00)	25,379.73	**27,070.17**	24,701.72
Qmat-S			4.62(0.00)	4.49(0.00)	25,333.07	27,143.19	24,607.08
Qmat-DS			3.40(0.13)	3.27(0.20)	25,407.38	27,895.68	**24,409.38**
Qmat-K			4.77(0.00)	4.64(0.00)	25,360.40	27,250.31	24,602.40
Qmat-DB			4.22(0.00)	4.09(0.01)	**25,305.49**	27,135.56	24,571.50
Qmat-DS-H			**3.03(0.47)**	**2.92(0.66)**	25,406.04	27,435.57	24,592.04
Qmat-E		ACDM	4.51(0.00)	4.56(0.00)	25,498.00	**27,073.76**	24,866.00
Qmat-S			5.67(0.00)	5.02(0.00)	25,512.47	27,118.14	24,868.46
Qmat-DS			5.24(0.00)	5.11(0.00)	25,476.82	27,157.29	**24,802.82**
Qmat-K			5.05(0.00)	4.92(0.00)	25,508.08	27,128.72	24,858.16
Qmat-DB			5.76(0.00)	5.62(0.00)	25,526.39	27,137.05	24,880.40
Qmat-DS-H			**4.50(0.00)**	**4.46(0.00)**	**25,466.52**	27,142.09	24,816.52
Qmat-E		RRUM	**5.04(0.00)**	5.52(0.00)	25,484.14	**27,059.89**	24,852.14
Qmat-S			5.78(0.00)	5.64(0.00)	25,486.80	27,092.48	24,842.80
Qmat-DS			6.17(0.00)	6.08(0.00)	25,597.67	27,278.14	24,923.68
Qmat-K			5.35(0.00)	5.87(0.00)	**25,469.00**	27,089.63	24,819.00
Qmat-DB			5.47(0.00)	**5.34(0.00)**	25,476.66	27,087.32	24,830.66
Qmat-DS-H			5.29(0.00)	5.46(0.00)	25,477.20	27,122.77	**24,817.20**
Qmat-E	Simulated dataset (*N* = 1000)	G-DINA	3.75(0.03)	3.76(0.03)	23,592.35	25,255.74	22,914.36
Qmat-S			2.80(0.96)	2.72(1.00)	23,532.17	25,313.32	22,806.16
Qmat-DS			4.08(0.00)	4.09(0.01)	23,604.22	26,052.69	**22,606.22**
Qmat-K			**2.52(1.00)**	**2.46(1.00)**	**23,503.60**	25,363.26	22,745.60
Qmat-DB			2.56(1.00)	2.68(1.00)	23,511.64	**25,312.42**	22,777.64
Qmat-DS-H			3.60(0.06)	3.61(0.06)	23,520.47	25,517.52	22,706.48
Qmat-E		ACDM	4.52(0.00)	4.52(0.00)	23,678.30	25,228.83	23,046.30
Qmat-S			**2.60(1.00)**	2.71(1.00)	**23,583.27**	**25,163.25**	**22,939.28**
Qmat-DS			6.05(0.00)	5.96(0.00)	23,761.85	25,415.42	23,087.84
Qmat-K			5.27(0.00)	5.29(0.00)	23,670.06	25,264.76	23,020.06
Qmat-DB			6.08(0.00)	5.97(0.00)	23,768.12	25,353.00	23,122.12
Qmat-DS-H			2.71(1.00)	**2.69(1.00)**	23,604.97	25,224.20	22,944.98
Qmat-E		RRUM	4.59(0.00)	4.59(0.00)	23,698.59	25,249.12	23,066.58
Qmat-S			**2.59(1.00)**	**2.69(1.00)**	23,588.70	**25,168.67**	22,944.70
Qmat-DS			6.28(0.00)	6.21(0.00)	23,727.61	25,381.19	23,053.62
Qmat-K			5.24(0.00)	5.27(0.00)	23,666.44	25,261.13	23,016.44
Qmat-DB			6.14(0.00)	6.05(0.00)	23,738.35	25,323.23	23,092.28
Qmat-DS-H			3.28(0.19)	3.22(0.23)	**23,586.07**	25,205.29	**22,926.06**

Note. The best-fitting values are presented in **bold.**

**Table 9:** Parameter stability comparison of six Q-matrices.

Item s	Qmat-E		Qmat-S		Qmat-DS		Qmat-K		Qmat-DB		Qmat-DS-H	
	*g*	*s*	*g*	*s*	*g*	*s*	*g*	*s*	*g*	*s*	*g*	*s*
Item 1	0.458	0.133	0.538	0.113	0.526	0.120	0.495	0.131	0.585	0.104	0.323	0.087
Item 2	0.000	0.062	0.000	0.000	0.371	0.204	0.000	0.157	0.000	0.000	0.268	0.190
Item 3	0.494	0.137	0.391	0.148	0.458	0.170	0.455	0.144	0.539	0.148	0.379	0.115
Item 4	0.441	0.045	0.348	0.206	0.651	0.145	0.529	0.075	0.515	0.193	0.283	0.093
Item 5	0.357	0.281	0.387	0.336	0.335	0.232	0.375	0.238	0.433	0.297	0.181	0.000
Item 6	0.344	0.049	0.000	0.269	0.000	0.237	0.449	0.092	0.000	0.225	0.235	0.150
Item 7	0.000	0.371	0.183	0.319	0.000	0.218	0.181	0.361	0.054	0.426	0.231	0.191
Item 8	0.000	0.000	0.270	0.000	0.234	0.256	0.201	0.190	0.301	0.181	0.252	0.233
Item 9	0.000	0.247	0.197	0.196	0.226	0.000	0.184	0.000	0.110	0.000	0.212	0.163
Item 10	0.445	0.073	0.000	0.088	0.552	0.102	0.000	0.103	0.239	0.121	0.000	0.120
Item 11	0.285	0.314	0.189	0.173	0.191	0.160	0.161	0.198	0.167	0.000	0.233	0.186
Item 12	0.365	0.061	0.000	0.037	0.242	0.038	0.000	0.031	0.000	0.011	0.216	0.034
Item 13	0.221	0.040	0.000	0.049	0.097	0.021	0.274	0.000	0.269	0.039	0.080	0.037
Item 14	0.204	0.007	0.137	0.012	0.227	0.006	0.214	0.010	0.183	0.020	0.000	0.005
Item 15	0.215	0.042	0.161	0.000	0.312	0.058	0.182	0.068	0.152	0.053	0.241	0.000
Item 16	0.000	0.213	0.000	0.212	0.415	0.153	0.275	0.155	0.000	0.193	0.000	0.242
Item 17	0.015	0.273	0.067	0.253	0.000	0.273	0.000	0.230	0.047	0.324	0.073	0.155
Item 18	0.090	0.393	0.126	0.380	0.000	0.000	0.128	0.348	0.113	0.000	0.081	0.191
Item 19	0.113	0.128	0.000	0.105	0.000	0.000	0.000	0.132	0.381	0.000	0.000	0.083
Item 20	0.231	0.000	0.282	0.000	0.000	0.156	0.167	0.183	0.150	0.157	0.000	0.119
Mean	0.214	0.143	0.164	0.145	0.242	0.127	0.213	0.142	0.212	0.125	0.164	0.120
Mean SE	0.040	0.029	0.036	0.028	0.047	0.021	0.039	0.023	0.043	0.028	0.027	0.017

**Table 10:** Classification accuracy of the four Q-matrices.

Q-Matrices	Test Level	Attribute Level							
	P_(a)_	P_(a)A1_	P_(a)A2_	P_(a)A3_	P_(a)A4_	P_(a)A5_	P_(a)A6_	P_(a)A7_	P_(a)A8_
Qmat-E	0.570	0.855	0.940	0.830	0.890	0.838	0.962	0.874	0.884
Qmat-S	0.696	0.951	0.931	0.868	0.936	0.899	0.953	0.886	0.901
Qmat-DS	0.692	0.861	0.928	0.972	0.920	0.870	0.892	0.953	0.899
Qmat-K	0.674	0.877	0.925	0.881	0.863	0.937	0.978	0.947	0.893
Qmat-DB	0.709	0.999	0.948	0.947	0.904	0.891	0.950	0.900	0.878
Qmat-DS-H	0.712	0.880	0.930	0.951	0.940	0.917	0.981	0.928	0.898

**Table 11:** Number of attribute correlation pairs by magnitude across six Q-matrices.

Q-Matrices	High (>0.70)	Intermediate (0.20–0.70)	Low (<0.20)
Qmat-E	0	14	14
Qmat-S	0	8	20
Qmat-DS	0	10	18
Qmat-K	0	19	9
Qmat-DB	0	6	22
Qmat-DS-H	0	24	4

## Data Availability

The data presented in this study can be made available upon reasonable request from the corresponding author.

## Supplementary Materials

The following supporting information can be downloaded at: https://www.mdpi.com/article/10.3390/jintelligence14050079/s1, S1: Full prompts for developing GenAI-informed Q-matrices; S2: Full diagnostic test paper; S3: Output documentations example by Doubao model.

## Appendix A

### Tetrachoric Correlations Among Attributes Across Six Q-Matrices

**Table A1:** Tetrachoric correlations among attributes estimated by Qmat-E.

	A1	A2	A3	A4	A5	A6	A7	A8
A1	1							
A2	0.06	1						
A3	0.16	0.26	1					
A4	0.28	−0.02	0.1	1				
A5	0.49	−0.11	0.3	0.6	1			
A6	0.22	0.26	0.43	0.25	0.2	1		
A7	−0.23	0.07	0.25	0.08	−0.13	0.3	1	
A8	0.02	0.28	−0.09	0.02	−0.28	0.19	0.5	1

**Table A2:** Tetrachoric correlations among attributes estimated by Qmat-S.

	A1	A2	A3	A4	A5	A6	A7	A8
A1	1							
A2	0.1	1						
A3	0.16	0.24	1					
A4	0.09	0.07	0.14	1				
A5	0.13	−0.04	0.15	0.22	1			
A6	0.01	0.18	0.25	0.01	−0.05	1		
A7	0.03	0.07	−0.19	0.17	0.53	0.11	1	
A8	0.24	0.21	0.26	0.26	−0.25	0.18	0.17	1

**Table A3:** Tetrachoric correlations among attributes estimated by Qmat-DS.

	A1	A2	A3	A4	A5	A6	A7	A8
A1	1							
A2	0.05	1						
A3	−0.01	0.28	1					
A4	−0.02	−0.12	0.07	1				
A5	−0.11	0.14	0.26	0.34	1			
A6	0.37	0.03	0.16	0.32	0.31	1		
A7	0.14	0.08	0.12	0.14	−0.24	0.32	1	
A8	0.09	0.5	0.3	0.07	0.42	−0.08	0.17	1

**Table A4:** Tetrachoric correlations among attributes estimated by Qmat-K.

	A1	A2	A3	A4	A5	A6	A7	A8
A1	1							
A2	0.35	1						
A3	0.08	0.39	1					
A4	0.57	0.48	0.2	1				
A5	0.23	0.25	0.16	0.39	1			
A6	0.03	0.23	0.22	0.21	0.21	1		
A7	−0.22	0.15	0.41	0.42	0.24	0.5	1	
A8	0.44	0.22	0.07	0.24	0.14	0.18	0.17	1

**Table A5:** Tetrachoric correlations among attributes estimated by Qmat-DB.

	A1	A2	A3	A4	A5	A6	A7	A8
A1	1							
A2	0.22	1						
A3	0.08	0.12	1					
A4	0.08	−0.04	0.15	1				
A5	0.12	−0.22	0.01	−0.08	1			
A6	0.12	0.11	−0.04	0.14	0.21	1		
A7	0.34	0.53	0.09	0.1	−0.09	0.08	1	
A8	0.1	0.06	−0.06	0.26	0.11	0.28	−0.15	1

**Table A6:** Tetrachoric correlations among attributes estimated by Qmat-DS-H.

	A1	A2	A3	A4	A5	A6	A7	A8
A1	1							
A2	0.28	1						
A3	0.36	0.49	1					
A4	0.69	0.33	0.45	1				
A5	0.25	0.24	0.33	0.21	1			
A6	0.16	0.21	0.34	0.31	0.04	1		
A7	0.36	−0.20	0.38	0.61	0.29	0.17	1	
A8	0.29	0.49	0.24	0.23	0.58	0.27	0.34	1
