# Supplementary material for: Applying GenAI to Optimize Q-Matrix Construction for Cognitive Diagnostic Assessment in EFL Reading
Source: J Intell. 2026 May 5;14(5):79. doi: 10.3390/jintelligence14050079 (PMC13208814; doi:10.3390/jintelligence14050079)
Supplement: Supplementary file 1 [file jintelligence-14-00079-s001.zip › jintelligence-4193271-supplementary.pdf]

Supplementary Materials

S1. Full prompts for developing GenAI-informed Q-matrices

Prompt 1: You are an expert specialized in cognitive diagnostic assessment (CDA) and language testing. Your task is to perform a rigorous cognitive analysis of an EFL diagnostic reading test and construct a corresponding Q-matrix. The test targets learners at approximately CSE 5 to CSE 6. Your analysis must be based solely on the provided mate-rials and definitions, simulating the deductive reasoning process of a content expert.

Prompt 2: Please review and internalize the following operational definitions of eight cognitive attributes. These definitions will serve as the exclusive coding framework for your analysis. Later, you are going to code which attribute or attribute combinations each test item measures.

Definition of Eight reading subskills

A1：Referential inference：Inferring the meaning of the antecedents of pronouns such as "it", "them", "this", etc. based on previous textual information.

A2：Lexical inference：Inferring the meaning of an ambiguous or unfamiliar word through contextual clues (i.e., searching the surrounding textual information of such word for clues).

A3: Temporal inference：Inferring the order of a series of related events or sentences based on their logical inter-connectedness, the relevant textual information, or background knowledge.

A4：Causative inference：Inferring the causes or purposes of events or statements given in the text based on the textually implicit causal-relation, the relevant textual information, or background knowledge.

A5: Premise-conclusion inference: Inferring the conclusion of events or statements based on a series of premises explicitly stated in previous text, or background knowledge.

A6: Thematic inference: Inferring the main idea of a paragraph or the text based on a whole understanding of the textual information, or background knowledge.

A7: Understanding sentence literal meaning: Understand sentence structure and literal meaning using knowledge of lexis, syntax, or grammar.

A8: Understanding discourse literal meaning: Integrate the meaning of multiple sentences into an understanding of the relationship between sentences, paragraphs, or larger sections of texts.

Prompt 3: You will construct a binary Q-matrix. For each item, assign a value of '1' if and only if mastery of the specific attribute or attribute combinations is theoretically necessary for a test-taker to answer the item correctly. Assign a value of '0' if the attribute or attribute combinations are not necessary for a correct response.

Prompt 4: Now, please read Passage 1 carefully and answer item 1. You should briefly explain the cognitive processes required to answer this item correctly. Based on your answering process, code which attribute or attribute combinations item 1 measures. If you identify any new attributes, propose them with clear operational definitions and justify its necessity.

Prompt 5: Now, please read Passage 2 carefully and answer item 2. You should briefly

explain the cognitive processes required to answer this item correctly. Based on your answering process, code which attribute or attribute combinations item 2 measures. If you identify any new attributes, propose them with clear operational definitions and justify its necessity.

Prompt 6: Now, please read Passage 3 carefully and answer item 3. You should briefly explain the cognitive processes required to answer this item correctly. Based on your answering process, code which attribute or attribute combinations item 3 measures. If you identify any new attributes, propose them with clear operational definitions and justify its necessity.

Prompt 7: Now, please read Passage 4 carefully and answer item 4. You should briefly explain the cognitive processes required to answer this item correctly. Based on your answering process, code which attribute or attribute combinations item 4 measures. If you identify any new attributes, propose them with clear operational definitions and justify its necessity.

Prompt 8: Now, please read Passage 5 carefully and answer item 5. You should briefly explain the cognitive processes required to answer this item correctly. Based on your answering process, code which attribute or attribute combinations item 5 measures. If you identify any new attributes, propose them with clear operational definitions and justify its necessity.

Prompt 8: Now, please read Passage 6 carefully and answer item 6. You should briefly explain the cognitive processes required to answer this item correctly. Based on your answering process, code which attribute or attribute combinations item 6 measures. If you identify any new attributes, propose them with clear operational definitions and justify its necessity.

Prompt 9: Now, please read Passage 7 carefully and answer item 7 and item 8. You should briefly explain the cognitive processes required to answer this item correctly. Based on your answering process, code which attribute or attribute combinations item 7 and item 8 measures. If you identify any new attributes, propose them with clear operational definitions and justify its necessity.

Prompt 10: Now, please read Passage 8 carefully and answer item 9. You should briefly explain the cognitive processes required to answer this item correctly. Based on your answering process, code which attribute or attribute combinations item 9 measures. If you identify any new attributes, propose them with clear operational definitions and justify its necessity.

Prompt 11: Now, please read Passage 9 carefully and answer item 10. You should briefly explain the cognitive processes required to answer this item correctly. Based on your answering process, code which attribute or attribute combinations item 10 measures. If you identify any new attributes, propose them with clear operational definitions and justify its necessity.

Prompt 12: Now, please read the Passage carefully and answer item 11 to item15. You should briefly explain the cognitive processes required to answer this item correctly. Based on your answering process, code which attribute or attribute combinations item 11 to item 15 measures. If you identify any new attributes, propose them with clear operational definitions and justify its necessity

Prompt 13: Now, please read Passage 16 to 18 carefully and answer item 16 to 18. You should briefly explain the cognitive processes required to answer this item correctly. Based on your answering process, code which attribute or attribute combinations item 16 to 18 measures. If you identify any new attributes, propose them with clear operational definitions and justify its necessity.

Prompt 14: Now, please read Passage 19 to 20 carefully and answer item 19 to 20. You should briefly explain the cognitive processes required to answer this items correctly. Based on your answering process, code which attribute or attribute combinations item 19 to 20 measures. If you identify any new attributes, propose them with clear operational definitions and justify its necessity.

S2. Full diagnostic test paper

第一部分

说明：本部分设有 9 个段落，共 10 道题目，文中可能的生词已用中文标出。请仔细阅读段落内容，在每道题后 A)、B)、C)、D)四个选项中选出正确答案。

Paragraph 1

When a comet (彗星) first becomes visible from Earth, it appears very small, but as it approaches the Sun, solar energy begins to vaporize(蒸发) the frozen gases, producing a glowing head called the coma. The size of the coma varies greatly from one comet to another. Extremely rare ones exceed the size of the Sun, but most approximate the size of Jupiter(木星). Within the coma, a small glowing nucleus(内核) with a diameter of only a few kilometers can sometimes be ***detected.*** As comets approach the Sun, some develop a tail that extends for millions of kilometers. Despite the enormous size of their tails and comas, comets are relatively small members of the solar system.

1. The word ***detected*** in the passage is closest in meaning to______.

   A) noticed　　B) created　　C) expanded　　D) displayed

Paragraph 2

Over the last several decades, environmental specialists have proposed various strategies aimed at slowing down the process of deforestation(森林开伐) in developing countries. Many of these proposals are indeed valuable ideas in that they are realistic attempts to address some of the causes of deforestation, such as farming, cattle ranching, and commercial logging. All of ***them*** rely on government involvement of some kind.

2. What does the word "***them***' refer to______?

   A) The causes of deforestation　　B) Decades

   C) Proposals　　D) Developing countries

Paragraph 3

Finally, international agreements usually involve the exchange of monetary (财政的) aid in return for government action to protect its forests. One such plan seeks to help pay a nation's debt in exchange for restrictions on certain kinds of activities in rainforests. ***This*** is appealing for a poor country such as Brazil, which has an international debt of $ 160 billion.

3. The word "***This***" in the paragraph refers to_______?

   A) a poor country　　B) a nation's debt

C) an international agreement D) an obligation

Paragraph 4

The statuses (身份) we assume often vary with the people we encounter, and change throughout life. Most of us can, at very high speed, assume the statuses that various situations require. Much of social interaction consists of identifying and selecting among appropriate statuses and allowing other people to assume their statuses in relation to us. This means that we fit our actions to those of other people based on a constant mental process of appraisal(评估) and interpretation. Although some of us find the task more difficult than others, most of us perform ***it*** rather effortlessly.

4. In the last sentence of this paragraph, the pronoun "***it***" refers to "________".

   A) constant mental process

   B) identification of other people's statuses

   C) selecting one's own statuses

   D) fitting our actions to those of other people appropriately

Paragraph 5

What morphological (形态的) and ecophysiological(生理生态的) characteristics determine the species composition(构成) and abundance in succession? In general, nitrogen-fixing (固氮的) plants (plants that can make use of atmospheric nitrogen) are important early succession species in primary succession because nitrogen is not derived from the weathering(风化) of rock and little or no organic matter is present in the soil. Weedy (似杂草的) plants are common early successional species because of their rapid growth and high reproductive rates, while stress-tolerant (耐拥挤的) species are common late successional species.

5. This paragraph supports the idea that nitrogen-fixing plant species are heavily represented in the early stages of a primary succession because they________.

   A) generally grow more rapidly than other plants.

   B) do not require soil that contains nitrogen.

   C) tend to be more stress tolerant than other plants.

   D) tend to be less weedy than other plants.

Paragraph 6

The great land estates (财产) of English society largely remained intact (完整的) and many wealthy land owners aggressively increased the size of their holdings, a precondition (前提) for increased productivity. Marriages between the children of the landowners also increased the size of land estates. Primogeniture (长子继承权) helped prevent land from being subdivided(再分割). Younger sons of independent owners left the family and went to find other respective locations. Larger farm contributed more to commercialized(商业化) farming at the time when an expanding population pushed up demand and prices. Farmland owners turned part of their land into pasture (牧场) land for sheep in order to adapt to developing woolen (羊毛的) trade.

6. Why does the author mention that farmland owners turned part of their land into pasture land for sheep?

A) To explain why the younger sons of land owners had to leave the family land and find other jobs

B) To provide an example of a way in which large farms were eager to improve their business

C) To explain how the practice of primogeniture worked

D) To describe a strategy land owners used to give their lands from being subdivided

Paragraph 7

Yet this most fundamental standard of historical periodization(时期) conceals (隐藏) a host of paradoxes. Nearly every movie theater, however modest, had a piano or organ to provide musical accompaniment to silent pictures. In many instances, spectators(观众) in the era before recorded sound experienced elaborate (精心制作的) aural (听觉的) presentations alongside movies' visual images, from the Japanese benshi (narrators) crafting multivoiced dialogue narratives to original musical compositions performed by symphony-size orchestras (管弦乐队) in Europe and the United States. In Berlin, for the premiere performance outside the Soviet Union of The Battleship Potemkin, film director Sergei Eisenstein worked with Austrian composer Edmund Meisel (1874-1930) on a musical score (乐谱) matching sound to image; the Berlin screenings with live music helped to bring the film its wide international fame.

7. The paragraph suggests which of the following about Eisenstein's film The Battleship Potemkirf?

   A) The film was not accompanied by sound before its Berlin screening.

   B) The film was unpopular in the Soviet Union before it was screened in Berlin.

   C) Eisenstein's film was the first instance of collaboration between a director and a composer.

   D) Eisenstein believed that the musical score in a film was as important as dialogue.

8. Why does the author mention "Japanese benshi" and "original musical compositions"?

   A) To suggest that audiences preferred other forms of entertainment to film before the transition to sound in the 1920's.

   B) To provide examples of some of the first sounds that were recorded for film.

   C) To show how the use of sound in films changed during different historical period.

   D) To indicate some ways in which sound accompanied film before the innovation of sound films in the late 1920s.

Paragraph 8

Ants are probably the most frequent and certainly the most persistent defenders of plants. Since the highly active worker ants require a great deal of energy, plants exploit (利用) this need by providing extrafloral(在花外的) nectar (花蜜) that supplies ants with abundant energy. To return this favor, ants guard the nectaries, driving away or killing

intruding (入侵的) insects that might compete with ants for nectar. Many of these intruders are herbivorous (食草的) and would eat the leaves of the plants.

9. What can be inferred from this paragraph about the ants that are attracted to the extrafloral nectaries?

   A) They do not eat the leaves of the plants that produce extrafloral nectar.

   B) They live almost entirely on extrafloral nectar.

   C) They spend most of their energy guarding extrafloral nectaries.

   D) They frequently fight among themselves over extrafloral nectar.

Paragraph 9

Fractures (骨折) and other cut marks are another source of evidence. Right-handed soldiers tend to be wounded on the left. The skeleton (骨架) of a 40- or 50-year-old Nabatean (那巴顿的) warrior, buried 2,000 years ago in the Negev Desert, Israel, had multiple healed fractures to the skull (颅骨), the left arm, and the ribs (肋骨).

10. Which of the following statements about fractures and cut marks can be inferred from this paragraph?

   A) Fractures and cut marks caused by right-handed soldiers tend to occur on the right side of the injured party's body.

   B) The right arm sustains more injuries because, as the dominant arm, it is used more actively.

   C) In most people, the left side of the body is more vulnerable to injury since it is not defended effectively by the dominant arm.

   D) Fractures and cut marks on fossil humans probably occurred after death.

第二部分

说明：请仔细阅读以下关于 *disposable cameras*(**拍立得相机**)的文章．在给出的 A--F 六个选项中，为每一段落选出最合适的标题。(注：其中一个标题为多余选项)

11. Paragraph 1 ____________
12. Paragraph 2 ____________
13. Paragraph 3 ____________
14. Paragraph 4 ____________
15. Paragraph 5 ____________

A. Photos and the social media
B. Old and new ways with photos
C. My solution to the problem
D. Prevalence of cameras
E. Disadvantages of the new way of photo-taking
F. Advantages of using disposable camera

Disposable Cameras Are the Future

Paragraph 1

Taking and sharing photographs is easier than ever. Cameras built into our phones, computers, tablets, and iPads allow us to send out photographs to each other. There was a time when you'd print your photos and file them away into photo albums.

Paragraph 2

Not only do we have what seems like an endless amount of space to store photos on our devices, but we also have the Internet, and specifically, social media. Social networks such as WeChat allow us to post our pictures and albums to share with friends and family.

People can now even become famous solely based on the photos that they post to these social networks.

Paragraph 3

While you could argue this easy ability to store, share and enjoy photos is fantastic, it also could be said that photographs have lost some of their meaning. I have over 1,000 pictures on my smartphone and several thousand stored on my computer. Sometimes, however, I'll be in the middle of taking or posting a photo and think: is this really something worth doing?

Paragraph 4

Even though I'm guilty of occasionally taking meaningless and boring pictures, a couple of years ago I decided to find a way to make my photographs more meaningful. How did I do this? Disposable cameras! When I go on a trip, I always try to take a disposable camera. As you can only take 27 pictures, you really think about the photographs you're taking. It's also exciting that you can't see the photographs straight away. And finally, it's so much fun to get the pictures developed and look through them again.

Paragraph 5

I'll admit that I couldn't live without social media or taking photographs on my smartphone, but I do think taking photographs has become less meaningful. That's why I'll continue using disposable cameras. It's a great way to capture real memories and make our photographs more meaningful.

第三部分

说明：本部分共 3 道题目，每道题目包含若干被打乱顺序的句子。请仔细阅读，并根据你的理解将打乱的句子重新排序，形成通顺的段落。请按照正确的排序，依次选出相应的句子编号。

16.

a. Within the first month, perceptual (感知的) abilities are still weak, and images more than a foot away are still out of focus.

b. By the age of about four months, a baby will be able to focus more clearly on a person moving around the room.

c. From the age of one week, a patterned surface (e.g. stripes (线条) or circles) is looked at more often than a colored plain surface.

d. Thereafter, visual abilities develop rapidly.

e. All infants are born with the ability to make certain perceptual distinctions and respond to them.

This paragraph should be re-ordered in the following way:

1._______2._______3._______4._______5._______

17.

a. The most important factor is the rise of more complex state-based societies, from traditional societies to the nation-states of today.

b. The fighting of war does not derive directly from human aggression(侵略).

c. From that time onwards, thousands of men fighting in regiments (军团) met on the fields of battle.

d. Armies were established and military discipline (军纪) introduced.

e. The origins of war must be traced to other factors.

f. With the development of more complex societies, things changed.

This paragraph should be re-ordered in the following way:
1._______2._______3._______4._______5._______6._______

18.

a. More than 100,000 wells now tap (开发) the Ogallala.

b. The ensuing (随后的) rapid expansion of irrigation agriculture (农业灌溉), especially from the 1950s onward, transformed the economy of the region.

c. The first wells were drilled into the Ogallala (奥加拉拉，地名) during the drought years of the early 1930s.

d. Modern irrigation devices, each capable of spraying (喷洒)4.5 million liters of water a day, have produced a landscape dominated by geometric (几何的) patterns of circular green islands of crops.

This paragraph should be re-ordered in the following way:
1._______2._______3._______4._______

第四部分

说明：本部分包含两道短问答题，请仔细阅读短文，推测目标词在该语境下的含义。

19.

A small amount of alcohol can result in changes in a person's mood and behaviour. A blood alcohol level of about .05% can make a person feel a sense of release from tension and inhibitions (拘束感). This mild ***euphoria*** is the aim of many people who drink moderately (适度地). However, this feeling of happiness drops as the alcohol level increases; there is an increasing loss of control because alcohol acts as a depressant (镇静剂) on functions of the brain.

请推测目标词“euphoria”的含义：

____

____

20.

The modem world is characterized by an increase in the means of communication and in opportunities to come into contact with other people. Yet in spite of all these things, there are many people who suffer from loneliness. This experience is painful and may serve as a source for a whole range of negative emotions such as self-pity, fear, timidity (胆小), anger, aggression and depression(抑郁). Such emotions can in turn lead to all kinds of destructive (破坏性的) behaviour which may ***sever*** a person's contact with other people

even more. Being cut off from social contact in this way serves to place such people in a vicious (残酷的) circle of ever-increasing loneliness.

请推测目标词“sever”的含义:

____

____

S3. Output documentations example by Doubao model

1) Pre-Analysis Boundaries & Theoretical Basis

- Target Population: EFL learners at CSE 5–CSE 6 (China’s Standards of English Language Ability)
  - ✧ CSE 5: Mastery of intermediate-to-advanced reading; able to process long expository/argumentative texts, locate key details, infer contextual meaning, and grasp basic discourse logic.
  - ✧ CSE 6: Advanced reading proficiency; able to interpret academic texts, analyze complex argumentation, integrate cross-paragraph information, and identify authorial stance & rhetorical purpose.
- Analytical Principle: Deductive expert reasoning; all cognitive attributes are discrete, measurable, non-redundant, and aligned with EFL reading construct & CSE 5–6 descriptors.
- Test Domain: Diagnostic EFL reading test (core item types: factual detail, lexical inference, syntactic parsing, main idea, discourse logic, cross-text integration, implicit reasoning, attitude/rhetorical analysis).

2) Based on your provided operational definitions, I have rigorously internalized the eight cognitive attributes and established a **standardized coding framework** for subsequent test item analysis. Below is the refined

attribute definition and coding rule set, which will be used exclusively for all subsequent item coding.

Part 1: Refined & Internalized Cognitive Attribute Definitions

Each attribute is defined with clear operational boundaries to ensure consistent and accurate coding, with core features highlighted for distinction:

Attribute	Core Name	Operational Definition (Internalized)	Key Distinguishing Features
**A1**	Referential Inference	Infer the specific referent of pronominal antecedents (e.g., *it, them, this, that*) by retrieving and matching explicit textual information from the preceding context.	Focus **exclusively on pronoun reference**; no lexical or logical reasoning involved.
**A2**	Lexical Inference	Deduce the meaning of ambiguous/unfamiliar words by identifying and analyzing **contextual clues** (synonyms, antonyms, examples, definitions, or	Focus **exclusively on word meaning deduction**; tied to the target word's textual context.

Attribute	Core Name	Operational Definition (Internalized)	Key Distinguishing Features
		restatements) in the surrounding text of the target word.	
**A3**	Temporal Inference	Determine the chronological order of a sequence of related events/sentences by analyzing their logical interconnections, explicit temporal cues (e.g., time adverbs), or background knowledge.	Focus **on time/sequence logic**; requires ordering of events.
**A4**	Causative Inference	Identify the implicit causal relationship (cause-effect, purpose-result) between events/statements in the text, or infer the	Focus **on causal/purposive logic**; links "why" to "what happened".

Attribute	Core Name	Operational Definition (Internalized)	Key Distinguishing Features
		cause/purpose of an event using textual clues and background knowledge.	
**A5**	Premise-Conclusion Inference	Derive a logical conclusion from a set of **explicitly stated premises** in the text, combined with background knowledge (no need for implicit premise extraction).	Focus **on deductive conclusion drawing**; premises are explicitly given in the text.
**A6**	Thematic Inference	Synthesize all relevant textual information (across sentences/paragraphs) to generalize the **main idea** of a single paragraph or the entire text (not just a single detail).	Focus **on holistic theme synthesis**; requires summarization of core ideas.

Attribute	Core Name	Operational Definition (Internalized)	Key Distinguishing Features
**A7**	Sentence Literal Meaning	Comprehend the explicit, literal meaning of a single sentence by applying lexical, syntactic, or grammatical knowledge (no inference beyond the sentence itself).	Focus **on intra-sentence literal comprehension**; no cross-sentence integration or inference.
**A8**	Discourse Literal Meaning	Integrate the meaning of multiple sentences to understand **explicit relationships** (e.g., addition, contrast, cause-effect) between sentences, paragraphs, or text sections (no inference of implicit meaning).	Focus **on inter-sentence/section explicit relationship understanding**; no deep inference or theme synthesis.

Part 2: Standardized Coding Rules

To ensure consistency and clarity in subsequent test item coding, the following rules will be strictly followed:

1. Single Attribute Coding: If an item measures only one cognitive attribute, use the attribute code directly (e.g., *A1*, *A2*).
2. Attribute Combination Coding: If an item measures two or more attributes simultaneously, connect the attribute codes with a plus sign (+) (e.g., *A2+A7*, *A6+A8*).
3. Priority of Core Attributes: For items with mixed attribute measurements, list the attributes in the order of their core contribution to the item's cognitive demand (e.g., an item requiring lexical inference first and sentence parsing second is coded *A2+A7*, not *A7+A2*).
4. Exclusivity: Only use the eight attributes above; no additional or alternative codes are allowed.

This framework has been fully internalized, and I am ready to conduct attribute coding for each test item in the EFL diagnostic reading test based on these definitions and rules.

3) **Item 1:**

Step 1: Cognitive Processes Required to Answer Item 1 Correctly

First, the test-taker must comprehend the literal meaning and grammatical structure of the target sentence: *Within the coma, a small glowing nucleus with a diameter of only a few kilometers can sometimes be detected.* This requires basic understanding of sentence syntax, lexical chunks, and plain sentential

meaning.

Second, the test-taker needs to locate the word *detected* in the passage, analyze its surrounding contextual clues (the context describes a glowing small nucleus inside the coma that can be perceived/identified), and infer the semantic meaning of the unfamiliar target word to match it with the closest synonym from the four options.

No reasoning about pronoun reference, time order, cause-effect, premise-conclusion logic, main theme, or cross-paragraph discourse relation integration is needed for this item.

Step 2: Attribute Coding (Strictly Based on the Provided 8 Definitions)

A2 (Lexical inference): 1 — The item core demands inferring the meaning of the word *detected* via surrounding contextual clues, which fully fits the definition of lexical inference.

A7 (Understanding sentence literal meaning): 1 — Mastery of single-sentence literal comprehension (syntax, basic vocabulary, sentence structure) is necessary to grasp the context for lexical guessing.

A1/A3/A4/A5/A6/A8: All 0 — These inferences or discourse-level skills are irrelevant and unnecessary for a correct response.

Final coding for Item 1: A2 + A7

Step 3: Evaluation of New Attributes

No new attributes need to be proposed.

Justification: The entire cognitive process of solving this item is fully

covered by the existing defined attributes A2 (lexical inference from context) and A7 (basic single-sentence literal understanding). There is an absence of unique, unclassifiable cognitive demands that cannot be categorized by the original 8 subskills.

4) **Item 2:**

Step 1: Cognitive Processes to Answer Item 2 Correctly

Locate the pronoun them in the final sentence of Paragraph 2: All of them rely on government involvement of some kind.

Understand the literal meaning and syntactic structure of the target sentence and the preceding sentences to retrieve the prior textual information (core nouns mentioned earlier in the context).

Trace back to find the logical antecedent of the pronoun them: the text repeatedly talks about these proposals / many of these proposals, so the pronoun refers to proposals.

Eliminate irrelevant options (causes of deforestation, decades, developing countries) with no need for lexical guessing, time sequencing, causal reasoning, theme summarizing, or cross-paragraph discourse integration.

Step 2: Attribute Coding (Strictly based on the 8 defined subskills)

A1 (Referential inference): 1-The core task is inferring the antecedent meaning of the pronoun them, which fully matches the definition of A1.

A7 (Understanding sentence literal meaning): 1- Basic comprehension of sentence structure and literal word meaning is necessary to identify the

prior referential context.

A2/A3/A4/A5/A6/A8: All assigned 0-these inference and discourse skills are unnecessary for a correct response.

Final coding for Item 2: A1 + A7

Step 3: Evaluation of New Attributes

No new attributes are required.

Justification: The entire cognitive demand of this pronoun-reference item is fully covered by the existing A1 (specialized for pronoun antecedent inference) and A7 (basic single-sentence literal comprehension). There are no unique cognitive operations that cannot be classified by the original eight subskills.

5) **Item 3:**

Step 1: Cognitive Processes to Answer Item 3 Correctly

1. Locate the demonstrative pronoun This in the target sentence at the end of Paragraph 3.
2. Trace back to the preceding context to retrieve the prior core content: the passage centers on *international agreements* that offer financial aid/debt relief in exchange for forest protection actions.
3. Confirm the logical antecedent of "This" — the whole arrangement of international agreements mentioned above, eliminating irrelevant options (poor country, a nation’s debt, an obligation).
4. Grasp the literal meaning and syntactic structure of the adjacent

sentences to accurately match the pronoun with its referent; no lexical guessing, time sequencing, causal reasoning, theme summarizing, or complex cross-paragraph discourse integration is required.

Correct answer: C

Step 2: Attribute Coding (Strictly following the 8 official definitions)

- A1 (Referential inference): 1 — The core task is inferring the antecedent of the pronoun *This*, which fully conforms to the definition of referential inference for pronouns.
- A7 (Understanding sentence literal meaning): 1 — Basic comprehension of single-sentence literal meaning and syntax is necessary to sort out contextual reference logic.
- A2/A3/A4/A5/A6/A8: All assigned 0 — these subskills are unnecessary for a correct response.

Final coding for Item 3: A1 + A7

Step 3: Evaluation of New Attributes

No new attributes need to be proposed.

Justification: All cognitive operations for solving this pronoun-reference item are fully covered by the existing A1 (pronoun antecedent inference) and A7 (intra-sentence literal comprehension). There is no unique cognitive demand that cannot be categorized by the original eight subskills.

6) **Item 4:**

Step 1: Answer & Cognitive Processes for Item 4

Correct Answer: D

1. Locate the pronoun *it* in the final sentence of Paragraph 4.
2. Trace back to identify the core task referred to by *it*: the prior key content is *we fit our actions to those of other people based on a constant mental process of appraisal and interpretation*.
3. Eliminate distracting options: A is the mental method supporting the task, B and C are partial small steps of social interaction, not the overall task that *it* stands for.
4. The whole process only requires figuring out the pronoun's antecedent and understanding the literal meaning of adjacent sentences; no lexical guessing, time sequencing, causal reasoning, theme generalization, or complex cross-paragraph discourse integration is needed.

Step 2: Attribute Coding (Strictly following the 8 official definitions)

- A1 (Referential inference): 1 — The core requirement is inferring the antecedent of the pronoun *it*, which fully matches the definition of referential inference for pronouns.
- A7 (Understanding sentence literal meaning): 1 — Mastery of single-sentence literal comprehension and syntactic structure is necessary to connect the pronoun with its original referent in the preceding text.
- A2, A3, A4, A5, A6, A8: All assigned 0 — these cognitive skills are not required for a correct response.

Final coding for Item 4: A1 + A7

Step 3: Evaluation of New Attributes

No new attributes are proposed.

Justification: All cognitive operations to solve this pronoun-reference item are fully covered by the existing A1 and A7. There is no unique or unclassifiable cognitive demand that cannot be categorized by the original eight subskills.

7) Item 5:

Step 1: Correct Answer & Cognitive Processes

Correct Answer: B

1. Locate the key causal sentence in Paragraph 5 about nitrogen-fixing plants in early primary succession.
2. Comprehend the literal meaning and syntactic structure of the target sentences: the text states nitrogen for early succession cannot come from rock weathering, and soil has almost no organic nitrogen; nitrogen-fixing plants can use atmospheric nitrogen on their own.
3. Infer the implicit cause for why these plants dominate early primary succession—they do not rely on nitrogen existing in the soil, which aligns with option B.
4. Eliminate distractors: A describes weedy plants; C refers to late-stage stress-tolerant species; D has no support in the text.
5. No pronoun reference, lexical guessing, time sequencing, main idea summarization, or cross-paragraph discourse integration is required.

Step 2: Attribute Coding (Strictly follow the 8 official definitions)

- A4 (Causative inference): 1 — The core task is inferring the *reason/cause* for the phenomenon described in the text, which fully matches the definition of causative inference.
- A7 (Understanding sentence literal meaning): 1 — Basic comprehension of single-sentence literal meaning and grammar is necessary to grasp the original causal clues in the text.
- A1/A2/A3/A5/A6/A8: All assigned 0 — these subskills are irrelevant and unnecessary for a correct response.

Final coding for Item 5: A4 + A7

Step 3: Evaluation of New Attributes

No new attributes are proposed.

Justification: All cognitive operations for solving this causal-reason question are fully covered by the existing A4 (causal inference) and A7 (intra-sentence literal comprehension). There is no unique cognitive demand that cannot be categorized by the original eight subskills.

8) Item 6:

Step 1: Correct Answer & Cognitive Processes

Correct Answer: B

1. Locate the key sentence in Paragraph 6: *Farmland owners turned part of their land into pasture land for sheep in order to adapt to developing woolen trade.*

2. Grasp the literal meaning of the sentence and the purpose marked by *in order to*, understanding this action is a business adjustment for the booming woolen trade under the background of commercialized farming.
3. Conduct purposive causal reasoning: infer the author's reason for mentioning this behavior — it serves as an example of large farms optimizing their commercial business.
4. Eliminate distractors: A links to younger sons leaving home (irrelevant); C refers to primogeniture mechanism (irrelevant); D relates to preventing land subdivision (function of primogeniture, not this action).
5. No pronoun reference, lexical guessing, time sequence sorting, main idea summarization or extra discourse integration is required.

Step 2: Attribute Coding (Strictly follow the 8 official definitions)

- A4 (Causative inference): 1 — The core task is inferring the purpose/cause of the landowners' behavior and the author's intention for citing it, which fully matches the definition of causative inference for reasons/purposes.
- A7 (Understanding sentence literal meaning): 1 — Basic comprehension of the target sentence's literal meaning and structural logic is necessary to capture the original purposive clue.
- A1/A2/A3/A5/A6/A8: All assigned 0 — these subskills are unnecessary for a correct response.

Final coding for Item 6: A4 + A7

Step 3: Evaluation of New Attributes

No new attributes are proposed.

Justification: The entire cognitive process of solving this purpose/cause question is completely covered by A4 (causative/purposive inference) and A7 (intra-sentence literal comprehension). There is no unique cognitive demand that cannot be classified by the original eight subskills.

9) **Item 7 and Item 8:**

Item 7 Analysis

Correct Answer

A

Cognitive Processes

1. Locate the specific information about *The Battleship Potemkin* in the paragraph; comprehend the literal meaning and syntactic details of the target sentence about its Berlin premiere and the newly created matching musical score (basic single-sentence understanding).
2. Extract explicit premises from the text: the matched musical score for image was specially produced for the premiere outside the Soviet Union in Berlin.
3. Draw a logical conclusion from these clear premises: the film had no such matched sound accompaniment before the Berlin screening.
4. Eliminate unsupported distractors (no evidence for popularity in the

Soviet Union, first collaboration, or comparison between music and dialogue).

5. No pronoun reference, lexical guessing, temporal ordering, thematic summarizing, or cross-paragraph discourse integration is required.

Attribute Coding (Strictly based on the 8 defined subskills)

- A5 (Premise-conclusion inference): 1 — Core reasoning to derive a conclusion from explicit textual premises.
- A7 (Understanding sentence literal meaning): 1 — Necessary to grasp the original premise information in the sentence.
- A1/A2/A3/A4/A6/A8: All 0

Final coding for Item7: A5 + A7

New Attribute Evaluation

No new attributes are needed. All cognitive operations are fully covered by the existing A5 and A7.

Item 8 Analysis

Correct Answer

D

Cognitive Processes

1. Locate the phrases *Japanese benshi* and *original musical compositions* in the context about silent movies; understand the literal meaning of the surrounding sentences describing elaborate aural presentations for silent films.

2. Infer the author's purpose/cause for providing these two examples: to illustrate various ways sound accompanied silent films before recorded sound films appeared.
3. Rule out wrong options: the examples are live on-site sound, not recorded sound; no comparison of audience preference or changes across historical periods is mentioned.
4. No pronoun inference, word meaning deduction, time sequence reasoning, conclusion drawing, or main idea generalization is required.

Attribute Coding (Strictly based on the 8 defined subskills)

- A4 (Causative inference): 1 — Core task: infer the author's purpose for citing the examples (inferring the reason behind the textual arrangement).
- A7 (Understanding sentence literal meaning): 1 — Required to understand the basic context of silent-film sound accompaniment.
- A1/A2/A3/A5/A6/A8: All 0

Final coding for Item8: A4 + A7

New Attribute Evaluation

No new attributes are proposed. The cognitive demands are completely captured by the pre-set A4 and A7; there are no unique unclassifiable skills.

10) **Item 9:**

Step 1: Correct Answer & Cognitive Processes

Correct Answer: A

1. Locate and comprehend the literal information in the paragraph: Plants provide extrafloral nectar for ants; ants drive away intruding herbivorous insects that eat the plants' leaves.
2. Extract explicit premises from the text: The leaf-eating pests are the intruders chased by ants; ants are the plant's guardians rewarded with nectar, not herbivorous intruders.
3. Draw a logical conclusion from the stated premises: The ants attracted by extrafloral nectaries do not eat the leaves of these plants.
4. Eliminate distractors: B is overstated (no evidence ants live *entirely* on the nectar); C is unsupported (no mention of spending *most energy* on guarding); D has no relevant content in the passage.
5. No pronoun reference, lexical inference, temporal ordering, causal purpose analysis for author's writing, or thematic summarization is required.

Step 2: Attribute Coding (Strictly following the 8 official definitions)

- A5 (Premise-conclusion inference): 1 — Core cognitive demand: deriving a valid conclusion based on explicitly stated textual premises.
- A7 (Understanding sentence literal meaning): 1 — Necessary to grasp the basic literal content of the cooperative relationship between ants and plants.
- A1/A2/A3/A4/A6/A8: All assigned 0

Final coding for Item 9: A5 + A7

Step 3: Evaluation of New Attributes

No new attributes are proposed.

Justification: The entire reasoning process relies solely on premise-conclusion inference (A5) and basic single-sentence literal comprehension (A7). There are no unique cognitive operations that cannot be classified by the original eight predefined subskills.

11) Item 10:

Step 1: Correct Answer & Cognitive Processes

Correct Answer: C

1. First, comprehend the literal meaning of the core sentences in Paragraph 9: the text states right-handed soldiers tend to receive wounds on the left side of the body, and an ancient warrior’s skeleton shows healed fractures on the left skull, left arm and ribs. This requires basic single-sentence literal understanding of facts (A7).
2. Extract the explicit premises from the given information: dominant right hand leads to left-side vulnerability to injury in combat.
3. Conduct premise-conclusion reasoning to generalize the logical inference: the left body is more easily injured because it lacks effective defense from the dominant hand, which matches option C.
4. Eliminate wrong options: A reverses the injury side; B has no supporting evidence in the text; D is contradicted by "healed fractures" (injuries occurred during life, not after death).

5. No pronoun reference, lexical guessing, temporal sequencing, author's purposive causal analysis, thematic summarization, or cross-sentence discourse integration is required.

Step 2: Attribute Coding (Strictly following the 8 official definitions)

- A5 (Premise-conclusion inference): 1 — Core task: draw a generalized logical conclusion based on explicitly stated textual premises.
- A7 (Understanding sentence literal meaning): 1 — Necessary to grasp the original factual premises in the text.
- A1/A2/A3/A4/A6/A8: All assigned 0

Final coding for Item 10: A5 + A7

Step 3: Evaluation of New Attributes

No new attributes are proposed.

Justification: All cognitive reasoning for this inferential item is fully covered by the existing A5 (premise-conclusion deduction) and A7 (intra-sentence literal comprehension). There are no unique cognitive demands that cannot be classified by the predefined eight subskills.

12) Item 11 to Item 15:

Item 11–15 Comprehensive Analysis (Disposable Cameras Passage)

Reaffirmation of Fixed 8 Attribute Definitions

A1=Referential inference; A2=Lexical inference; A3=Temporal inference; A4=Causative inference; A5=Premise-conclusion inference; A6=Thematic inference (main idea summary); A7=Sentence literal meaning;

A8=Discourse literal meaning (multi-sentence integration). No new attributes will be added unless necessary.

Item 11: Paragraph 1

Correct Answer

B. Old and new ways with photos

Cognitive Processes

1. Grasp the literal meaning of every single sentence in Paragraph 1: modern easy photo-taking/sharing via electronic devices, plus the old practice of printing photos and storing them in albums (A7).
2. Integrate the logical relationship between all sentences in the paragraph to understand the contrast between past and present photo-taking customs (A8).
3. Synthesize the overall core content of the whole paragraph to induce its central theme—comparing old and new photo-taking ways (A6).
4. No pronoun reasoning, lexical guessing, time/ cause/conclusion reasoning is required.

Attribute Coding

A6 + A7 + A8

New Attribute Evaluation

No new attributes are needed; all cognitive demands are fully covered by existing defined subskills.

Item 12: Paragraph 2

Correct Answer

A. Photos and the social media

Cognitive Processes

1. Understand the literal meaning of sentences about large storage space for photos and photo-sharing via social platforms (A7).
2. Integrate cross-sentence information to clarify the close connection between photos and social media in the digital era (A8).
3. Summarize the paragraph's dominant theme focusing on the link between photos and social media (A6).
4. No other inference skills are involved.

Attribute Coding

A6 + A7 + A8

New Attribute Evaluation

No new attributes proposed.

Item 13: Paragraph 3

Correct Answer

E. Disadvantages of the new way of photo-taking

Cognitive Processes

1. Comprehend the literal content: convenient photo storage/sharing makes photos lose meaning, and many photos are worthless (A7).
2. Integrate sentence relations to capture the critical attitude toward

modern photo-taking (A8).

3. Induce the main idea that the paragraph talks about the drawbacks of the new photo-taking mode (A6).

Attribute Coding

A6 + A7 + A8

New Attribute Evaluation

No new attributes proposed.

Item 14: Paragraph 4

Correct Answer

C. My solution to the problem

Cognitive Processes

1. Master the literal meaning of sentences: the author decides to use disposable cameras to make photos meaningful, and explains specific practices (A7).
2. Integrate multi-sentence discourse content to understand the author's targeted method solving the problem of meaningless photos (A8).
3. Summarize the paragraph theme: introducing the author's personal solution to the existing problem (A6).

Attribute Coding

A6 + A7 + A8

New Attribute Evaluation

No new attributes proposed.

Item 15: Paragraph 5

Correct Answer

F. Advantages of using disposable camera

Cognitive Processes

1. Understand the literal expression: the author insists on using disposable cameras because they can record real and meaningful memories (A7).
2. Integrate the whole paragraph's discourse logic to highlight the value and strengths of disposable cameras (A8).
3. Generalize the core theme focusing on the advantages of disposable cameras (A6).

Attribute Coding

A6 + A7 + A8

New Attribute Evaluation

No new attributes proposed. All theme-summary and discourse-integration tasks are fully covered by A6, A7 and A8.

Summary of Unified Coding for 11–15

All paragraph title matching items rely on three necessary attributes:

1. A7: Basic single-sentence literal comprehension as the foundation;
2. A8: Integrating multiple sentences to grasp internal discourse relations;
3. A6: Thematic inference to summarize the paragraph's main idea for title matching.

No extra cognitive skills or new attributes are required for this set of items.

13) Item 16 to Item 18:

Analysis for Item 16–18 (Sentence Reordering Tasks)

Reaffirmation of the 8 Fixed Cognitive Attributes

A1=Referential inference; A2=Lexical inference; A3=Temporal inference (event/ time order); A4=Causative inference; A5=Premise-conclusion inference; A6=Thematic inference; A7=Sentence literal meaning; A8=Discourse literal meaning (integrate multi-sentence relationships).

Item 16

Correct Sentence Order

1.e → 2.c → 3.a → 4.b → 5.d

Cognitive Processes

1. Grasp the literal meaning of every single sentence about infants' visual development (A7), and identify clear time markers: birth ability, 1 week, 1 month, 4 months, thereafter.
2. Apply temporal inference (A3) to judge the chronological sequence of visual growth based on age and developmental stages.
3. Integrate the sequential logical relationship between all sentences to form a coherent developmental timeline (A8).
4. No pronoun reference, lexical guessing, causal reasoning or theme summarization is required.

Attribute Coding

A3 + A7 + A8

New Attribute Evaluation

No new attributes are needed. All time-order reordering demands are fully covered by A3, A7 and A8.

Item 17

Correct Sentence Order

1.b → 2.e → 3.f → 4.d → 5.c → 6.a

Cognitive Processes

1. Understand the literal meaning of each sentence about the origin and evolution of war (A7).
2. Recognize the implicit causal logical chain: war is not from human aggression → other factors matter → complex social development changed situations → armies were built → large-scale battles appeared → the core cause is the rise of nation-states (A4 causative inference).
3. Integrate progressive logical relationships across all sentences to build a coherent argumentative discourse (A8).
4. No time-sequence or lexical inference is the core demand here.

Attribute Coding

A4 + A7 + A8

New Attribute Evaluation

No new attributes are proposed. The logical reordering based on cause-effect and discourse connection is fully captured by the existing three

attributes.

Item 18

Correct Sentence Order

1.c → 2.b → 3.a → 4.d

Cognitive Processes

1. Comprehend the literal information of each sentence about the development of the Ogallala water project, and capture explicit time clues: early 1930s → from the 1950s onward → now → modern technology (A7).
2. Use temporal inference (A3) to arrange events strictly in historical chronological order of well drilling and irrigation development.
3. Integrate the time-based connection between sentences to form a clear developmental timeline of regional agriculture (A8).

Attribute Coding

A3 + A7 + A8

New Attribute Evaluation

No new attributes are necessary. All cognitive operations for this time-sequence reordering are completely covered by the predefined attributes.

14) Item 19 to Item 20:

Analysis for Item 19 & Item 20 (Contextual Vocabulary Inference)

Reaffirmed Fixed 8 Cognitive Attributes

A1=Referential inference; A2=Lexical inference (contextual word meaning

deduction); A3=Temporal inference; A4=Causative inference; A5=Premise-conclusion inference; A6=Thematic inference; A7=Understanding sentence literal meaning; A8=Understanding discourse literal meaning.

Item 19

Step 1: Inferred Meaning of *euphoria*

A strong feeling of happiness, relaxation and pleasant excitement; mild joy and comfort brought by drinking a small amount of alcohol.

(简洁释义：幸福感；愉悦感；轻松狂喜的情绪)

Step 2: Cognitive Processes

1. Locate the target word *euphoria* and analyze surrounding contextual clues: the preceding content describes alcohol brings release from tension and inhibitions; the later text uses this feeling of happiness to refer back to the word directly.
2. Comprehend the literal meaning and logical connection of the adjacent sentences to capture the explanatory hints for the unfamiliar word (basic single-sentence understanding).
3. Deduce the semantic meaning of *euphoria* totally based on contextual clues, with no need for pronoun reference, time sorting, causal reasoning or main idea summarization.

Step 3: Attribute Coding

- A2 (Lexical inference): 1 (core cognitive demand for guessing word meaning via context)

- A7 (Sentence literal meaning): 1 (necessary to understand surrounding explanatory sentences)
- All other attributes: 0

Final coding: A2 + A7

Step 4: New Attribute Evaluation

No new attributes are proposed. The whole cognitive process fully conforms to the definition of A2 (lexical inference) assisted by basic A7 comprehension; no unclassifiable skills appear.

Item 20

Step 1: Inferred Meaning of *sever*

To cut off, break or separate completely; to end social contact and relationships between people.

(简洁释义：切断；断绝；割裂)

Step 2: Cognitive Processes

1. Locate the target word *sever* in the logical context: negative emotions lead to destructive behavior, which will make people's social connections worse and deepen loneliness in a vicious circle.
2. Extract causal and semantic clues from the sentence context to infer that destructive behavior will break off interpersonal contact.
3. Master the literal meaning of the whole sentence to ensure accurate understanding of the logical relationship behind the vocabulary; no extra complex inference or discourse integration is required.

Step 3: Attribute Coding

- A2 (Lexical inference): 1 (core task of deducing unknown word meaning from context)
- A7 (Sentence literal meaning): 1 (foundational for grasping contextual clues)
- All other attributes: 0

Final coding: A2 + A7

Step 4: New Attribute Evaluation

No new attributes are needed. The vocabulary inference task is completely covered by the pre-set A2 and A7, with no unique cognitive demands that cannot be categorized.

Overall Summary for 19–20

Both items are pure contextual lexical inference tasks; the unified coding is A2+A7, and no additional attributes are required in either case.

Synthesized Q-matrix (Qmat-DB):

Item	A1	A2	A3	A4	A5	A6	A7	A8	Item	A1	A2	A3	A4	A5	A6	A7	A8
Item 1	0	1	0	0	0	0	1	0	Item 11	0	0	0	0	0	1	1	1
Item 2	1	0	0	0	0	0	1	0	Item 12	0	0	0	0	0	1	1	1
Item 3	1	0	0	0	0	0	1	0	Item 13	0	0	0	0	0	1	1	1
Item 4	1	0	0	0	0	0	1	0	Item 14	0	0	0	0	0	1	1	1
Item 5	0	0	0	1	0	0	1	0	Item 15	0	0	0	0	0	1	1	1
Item 6	0	0	0	1	0	0	1	0	Item 16	0	0	1	0	0	0	1	1
Item 7	0	0	0	0	1	0	1	0	Item 17	0	0	0	1	0	0	1	1
Item 8	0	0	0	1	0	0	1	0	Item 18	0	0	1	0	0	0	1	1
Item 9	0	0	0	0	1	0	1	0	Item 19	0	1	0	0	0	0	1	0
Item 10	0	0	0	0	1	0	1	0	Item 20	0	1	0	0	0	0	1	0
